# Phenotypic heterogeneity in modeling cancer evolution

**DOI:** 10.1371/journal.pone.0187000

**Published:** 2017-10-30

**Authors:** Ali Mahdipour-Shirayeh, Kamran Kaveh, Mohammad Kohandel, Sivabal Sivaloganathan

**Affiliations:** 1 Department of Applied Mathematics, University of Waterloo, Waterloo, Canada; 2 Program for Evolutionary Dynamics, Harvard University, Cambridge, United States of America; 3 Center for Mathematical Medicine, Fields Institute, Toronto, Canada; University of Alabama at Birmingham, UNITED STATES

## Abstract

The unwelcome evolution of malignancy during cancer progression emerges through a selection process in a complex heterogeneous population structure. In the present work, we investigate evolutionary dynamics in a phenotypically heterogeneous population of stem cells (SCs) and their associated progenitors. The fate of a malignant mutation is determined not only by overall stem cell and non-stem cell growth rates but also differentiation and dedifferentiation rates. We investigate the effect of such a complex population structure on the evolution of malignant mutations. We derive exactly calculated results for the fixation probability of a mutant arising in each of the subpopulations. The exactly calculated results are in almost perfect agreement with the numerical simulations. Moreover, a condition for evolutionary advantage of a mutant cell versus the wild type population is given in the present study. We also show that microenvironment-induced plasticity in invading mutants leads to more aggressive mutants with higher fixation probability. Our model predicts that decreasing polarity between stem and non-stem cells’ turnover would raise the survivability of non-plastic mutants; while it would suppress the development of malignancy for plastic mutants. The derived results are novel and general with potential applications in nature; we discuss our model in the context of colorectal/intestinal cancer (at the epithelium). However, the model clearly needs to be validated through appropriate experimental data. This novel mathematical framework can be applied more generally to a variety of problems concerning selection in heterogeneous populations, in other contexts such as population genetics, and ecology.

## Introduction

Cancer can be thought of as a complex ecosystem in which not only tumor cells but also other cell types (phenotypes) may influence the overall health of an organism. Experimental results have recently shown that cancer cells may mimic the functional features of normal cells [1]. The most important features are associated with a small subpopulation of cells, namely the stem cells (SCs). SCs are defined to be cells with self-renewal capacity and pluripotency. For instance, they can replenish and regenerate the whole epithelial cell population in normal tissues. It has been proposed that cancer stem cells (CSCs) maintain invasive characteristics, such as (undesirable) multipotency and uncontrolled growth and tumor initiating capacity [2–6]. The non-stem/differentiated progenies of SCs are cells with specialized distinct functions within the organism. They are produced via a hierarchical division scheme. As the non-stem/differentiated cells (DCs) become more mature along the hierarchy, their replication potential decreases [7–10].

CSCs reside in small niches and manifest characteristics similar to somatic SCs [2]. In solid cancers, CSCs are usually imputed as a result of the expression of similar biomarkers as those used to identify SCs [11–15]. In colon cancer, the over—expression of the polycomb ring finger oncogene BMI1 leads to the down—regulation of proteins p16INK4a and p14ARF. These proteins override cellular proliferation restriction and generate cancer stem-like cells (SLCs) [6, 8, 16]. For mammary stem cells, CD44^+^ and CD24^−^ are reported as markers for stemness. In acute—myeloid leukemia (AML) CD34^+^ CD38^−^ cells are a leukemia—initiating subpopulation [6, 17].

Despite the new established dogma that cancer cells originate from a small niche of cells [6, 10, 18], a range of experiments have now investigated and reported on the cancer initiating capacity of committed progenitor cells [3, 6, 19]. In other words, non-SCs can undergo a dedifferentiation process and regain stemness (these cells are also called stem like cells). In breast cancer, epithelial—mesenchymal transition (EMT) factors have been implicated in the production of stem like cells from non—stem cells [20–22]. Gupta *et al* [23] have also observed that the epithelial non-stem cells with basal markers can convert to cells with stem cell markers (see also [21]). There are several other experimental observations supporting dedifferentiation of committed progenitor cells [6, 19, 24–26]. In addition, this dedifferentiation has been observed under certain microenvironmental conditions, in normal SCs [1, 27, 28]. In fact, it is becoming apparent that cellular dedifferentiation is activated in a number of organs to produce stem like cells in support of SCs in tissue regeneration [1, 6–8, 18, 19, 29, 30].

A variety of quantitative approaches have been utilized to investigate the effect of genetic heterogeneity in cancer initiation (and progression). How driver and passenger mutations contribute to the mutational heterogeneity of the tumor and its growth have been discussed in some details (e.g. see [31–37]).

In [38], by using an exact estimation based on Wright-Fisher model for colon cancer, the authors tried to find the expected time to tumorigenesis as well as the effect of relative selection pressure according to the population size and the mutation rate. For example, relationship between time to cancer and mutational heterogeneity drivers are discussed by Bozic *et al*. [34]. The role of passenger mutations in cancer progression has been addressed in [35]. Moreover, Durrett *et al*. discuss the influence of random variation in the fitness of driver mutants and intratumor heterogeneity [33]. However, an evolutionary approach, to phenotypic heterogeneity inside the tumor, co-evolution of various phenotypes with tumor microenvironment has received much less attention in the literature (see e.g. [39] for such an evolution).

Phenotypic heterogeneity inside a tumor relies on the notion of stem cell hierarchy and concept of cell-of-origin. To this end, much effort has been devoted to model sphere forming assays or tumoroids *in vitro*, which resemble the growth dynamics of tumor [40–43]. Deterministic and stochastic models have also been used to predict the population dynamics of tumor spheres starting from a single stem cell or a non-stem cell. (A)symmetric division scheme, dedifferentiation of progenitor population into stem cells are analyzed in the context of tumor sphere growth [42, 43]. These models are crudely applied to investigate the effect of tumor phenotypic/epigenetic heterogeneity on various clinical treatment regimens [42].

Phenotypic heterogeneity can also affect the risk of cancer. Pepper *et al*. presented a simple deterministic model of cell differentiation and somatic evolution. The authors studied the effect of mutational events as well as variations in replication and differentiation rates in a hierarchically structured population. They concluded that the structure of serial differentiation crucially suppresses the somatic evolution of tissues. Similarly Nowak and his co-authors studied a stochastic linear model of spatially organized stem and non-stem cells and showed that such a linear organization of diverse phenotypes leads to suppression of somatic evolution of malignancy [44].

Accumulation of mutations and mutational extinction time are both influenced by the stem cell hierarchical structure. To this end, Werner *et al*. discuss deterministic population dynamics of the stem cell hierarchy [45, 46]. Another simple hierarchical model for leukemic cells has been illustrated in [45, 47] to address the relevance of malignant mutations in various positions along the differentiation hierarchy. On the other hand, fixation time in the presence of dedifferentiation has been explored under a diffusion approximation [48] and also by studying replicator equations [49]. Shahriyari and Komarova [50] address the role of biclonal stem cell niche in decreasing the rate of the first double-hit-appearance. Similarly in [51], the rate of TSG production has been discussed regarding various probabilities for (a)symmetric division at different locations on a normal crypt. Then applying stochastic simulation and exact calculation, the authors show that optimal case to delay the onset of cancer may relate to the higher division rate at the top of the crypt.

Furthermore, division polarity which determines the type of division in individuals (symmetric vs. asymmetric division) has a wide variety of applications in biological systems. In the absence of selection pressure in a heterogeneous population of normal and mutants SCs, the crucial role of (a)symmetric division and time to fixation has been investigated in [52]. In this study, a mass-action model is taken into account. Moreover, Shahriyari *et al*. [53] demonstrate the rate of evolution in a simple hierarchical stem and non—stem cell population. The authors argue that stem cell symmetric division is preferred under natural selection for two-hit mutations. Furthermore, the role of migration in delaying cancer is investigated within a bi-compartmental stem cell niche [54]. A generalized multi-stage structure of the colonic/intestinal crypt has also been recruited to give an insight into the effect of subclonal heterogeneity, migration, immortality, and division polarity in carcinogenesis [55].

Despite the above modeling efforts to characterize evolutionary aspects of the hierarchical population structure of the cell-of-origin has been poorly studied. More precisely, there exist a lack for a general quantitative framework that accounts for selection and (genetic) mutation in phenotypically/epigenetic diverse population.

In this study, we consider a novel framework to study natural selection in heterogeneous populations where microenvironmentally induced plasticity exists. We analyze competition between normal and malignant populations which are genotypically different. Each of these groups divide into phenotypically different subpopulations (stem cell and non-stem subtypes). Due to homeostasis, the size of SC and DC subpopulations are assumed to remain constant. SCs can self—renew and replenish their own population or contribute to the DC population via differentiation events. Non-stem cells can also divide into DCs or dedifferentiate into SLC states. We investigate conditions for the successful selection of a malignant mutation in this complex population structure. Due to the plastic nature of the early malignant non-stem, there is a finite chance for an advantageous mutant to exit the non-stem cell group and become part of the SC niche. We derive exactly calculated results that predict the fixation probability of a mutant (either in the SC or DC subpopulations) to take over the system and establish a finite colony. We assume arbitrary population sizes and division rates and selection intensities as well as (de)differentiation rates. The exactly calculated results are in agreement with stochastic simulations in finite populations. We apply our findings to colorectal cancer—where plasticity has been observed under inflammatory environmental conditions. Our model predicts that dedifferentiation can confer a selection advantage for P53 mutants compared with the intrinsic/genetic fitness disadvantage of P53 mutants in the absence of inflammatory conditions (reported in [18]).

This novel mathematical framework can be applied more generally to a variety of problems. The method can also be used to calculate the chance of fixation in subdivided populations that arise in ecological models. For example in stepping stone meta-population models, differentiation and plasticity can be seen as (asymmetric) migration of individuals among islands. Evolution of multicellularity is yet another example. During evolution of simple multicellularity (for example Volvox algae [56, 57]) Cells with a higher proliferation capacity in a multicellular complex, reside with the more functional cells—for example those that give rise to a ‘swimming’/migration capability. The evolution of more complex functions can be modeled as a selection process inside each complex, with a heterogeneous population of cells, using the method we have developed.

The paper is organized as follows: in the Material and Methods section, the generalized Moran process is defined and the generating function method to calculate long-term fixation or extinction probabilities is presented. The replicator dynamics for the model is derived in the absence of random drift. In the Results section, we discuss the fixation probability as a function of stem cell self-renewal rate, differentiation rate and dedifferentiation rate. The phase diagrams for advantageous mutants are also presented towards the end of this section, where some applications of this study are states in the context of evolutionary and biological ecosystems. In the Discussion section, we summarize our findings and suggest some possible generalizations to characterize more heterogeneous environments.

The focus of our model is on the selection process of a single invading genotype. Generalization of the above framework to accumulation of several mutations, i.e. mutation-selection process, in a finite-population with a phenotypically diverse background is left for future studies.

## Materials and methods

### Generalized Moran process with (a)symmetric division and plasticity

Consider two populations of resident or wild type (type 1) and mutant or invader (type 2). Mutants are the result of an oncogenic mutation in the resident population. Each genotype is divided into phenotypically different subpopulations of stem cells (SC) and non-stem cells (DC). Stem cells can self-renew symmetrically where the offspring are both stem cells. They can differentiate (symmetrically or asymmetrically) to produce non-stem cell progenies of the same genotype. During a symmetric differentiation one stem cell produces two non-stem cell offsprings while within asymmetric differentiation the stem generates one stem cell and one non-stem cell.

We denote the probability of symmetric differentiation (per division) by ${\hat{u}}_{1},{\hat{u}}_{2}$ and asymmetric differentiation by ${\hat{v}}_{1},{\hat{v}}_{2}$. The overall probability of differentiation is $u_{1}={\hat{u}}_{1}+2{\hat{v}}_{1}$ and $u_{2}={\hat{u}}_{2}+2{\hat{v}}_{2}$. This is due to the fact that SCs can undergo symmetric division, producing two SCs (generation of two non-SCs can be considered as the result of two sequential asymmetric divisions in SCs per two unit times). The other possible scenario is associated with the asymmetric differentiation of SCs to produce one stem and one non-stem cell in two different ways: having the first daughter cell as stem cell and the second one as non-stem cell or vice versa). These events describe the coefficients 1 and 2 in the definition of *u*_1_ and *u*_2_.

Similarly the self-renewal probabilities is denoted by 1 − *u*_1_ and 1 − *u*_2_. The indexes 1 or 2 denote the corresponding probabilities for a wild type or mutant. The division rate of a normal (or mutant) stem cell is denoted by *r*_1_ (*r*_2_) respectively. Similarly, the division rates of non-stem cells are denoted by ${\tilde{r}}_{1}$ and ${\tilde{r}}_{2}$. For the sake of simplicity, one can assume that the death rates of normal and mutant cells (SCs and non-SCs) are all the same, in order to concentrate on the impact of changes in the reproduction rates on the evolution of malignancy. Similarly, we can change only the death rates and keep proliferation rates unchanged. These two different scenarios are symmetric in terms of their impact on the ultimate fate of the system.

**Fig 1:**
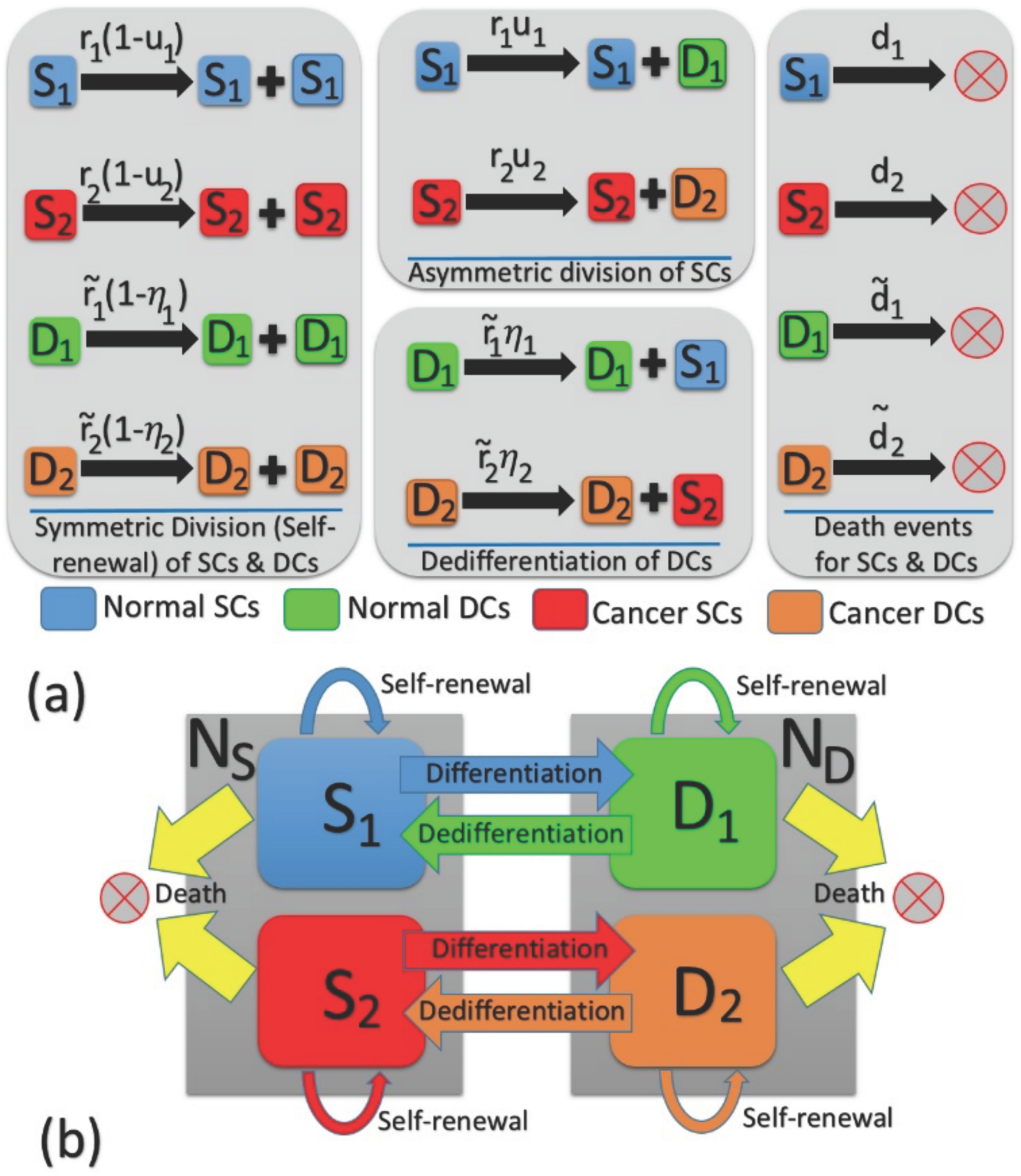
Phenotypic—Genotypic changes in individuals within a four–compartmental structure. We consider constant population sizes *N*_S_ and *N*_D_ for SCs and DCs respectively. With respect to the finite Markov chain, we consider a generalized model to take into account the competition between normal and malignant individuals in each of the SC and DC subpopulations. Differentiation and dedifferentiation events connect the selection dynamics between the two niches. In (a), all possible differentiation, dedifferentiation, and death events with their corresponding rates are represented. The SC-DC compartmental structure is depicted in (b) with the associated self—renewal and differentiation/plasticity possibilities.

For evolutionary dynamics we consider a birth-death (BD) Moran process as follows: We assume constant population sizes *N*_S_ and *N*_D_ respectively for SCs and DCs. Thus within each of the SC or DC compartments, there is competition between normal and malignant (SC or DC) individuals. At each time step, an individual is chosen to reproduce proportional to its fitness within the SC or DC compartments. If a normal (mutant) cell in the SC compartment is chosen to reproduce, its offspring replaces a randomly chosen cell in the stem cell compartment with probability 1 − *u*_1_ (or 1 − *u*_2_). Otherwise, with probability *u*_1_ (or *u*_2_), the (non-stem cell) offspring replaces a randomly chosen cell in the DC compartment. Similarly, if a non-stem cell is chosen to reproduce, its offspring replaces another cell in the non-stem cell compartment with the probability 1 − *η*_*i*_ (*i* = 1, 2). Alternatively, the offspring can dedifferentiate into a stem-like cell and replace a randomly chosen individual in the stem cell compartment with a rate *η*_1_ (or *η*_2_) denote the dedifferentiation probability for normal (mutant) DCs (see Table 1 for brief definitions of parameters and Fig 1 for the dynamics of the considered model). For simplicity we assumed death rates of all types to be equal and set this to unity.

**Table 1:** Model parameters.

Notation	Description
*N*_S_, *N*_D_	Total number of stem and non-stem cells
*r*_1_, *r*_2_	Net reproduction rate of wild type and mutant stem cells
${\tilde{r}}_{1},{\tilde{r}}_{2}$	Net reproduction rate of wild type and mutant non-stem cells
*u*_1_, *u*_2_	Asymmetric differentiation rate of normal and mutant stem cells
*η*_1_, *η*_2_	Dedifferentiation rate of normal and mutant non-stem cells

The above dynamics models the differentiation mechanism with an *effective* asymmetric division with the probabilities *u*_1_, *u*_2_. Thus in the following we use the terms differentiation (of stem cells) and asymmetric division interchangeably.

The above Moran process can be written as a continuous time process (1/*N* is the duration of each time step for *N* = *N*_S_ + *N*_D_)

$$\begin{array}{c} \frac{1}{N}\frac{\partial p(n_{S},n_{D};t)}{\partial t}=W_{S}^{+}\left(n_{S}-1,n_{D}\right)\;p\left(n_{S}-1,n_{D};t\right)+W_{S}^{-}\left(n_{S}+1,n_{D}\right)\;p\left(n_{S}+1,n_{D};t\right)\\ \;+\;W_{D}^{+}\left(n_{S},n_{D}-1\right)\;p\left(n_{S},n_{D}-1;t\right)+W_{D}^{-}\left(n_{S},n_{D}+1\right)\;p\left(n_{S},n_{D}+1;t\right)\\ \;-\;(W_{S}^{+}\left(n_{S},n_{D}\right)+W_{D}^{+}\left(n_{S},n_{D}\right)+W_{S}^{-}\left(n_{S},n_{D}\right)+W_{D}^{-}\left(n_{S},n_{D}\right))\;p\left(n_{S},n_{D};t\right). \end{array}$$ (1)

where *p*(*n*_S_, *n*_D_;*t*) denotes the probability of having *n*_S_ mutant stem cells and *n*_D_ mutant non-stem cells, at time *t* (given $n_{S}^{0}$ and $n_{D}^{0}$ at *t* = 0). The population of normal cells are given by *N*_S_ − *n*_S_ and *N*_D_ − *n*_D_ correspondingly. The probabilities $W_{S}^{\pm }$ and $W_{D}^{\pm }$ are the transition probabilities corresponding to an increase or decrease by one in the number of mutant SCs and DCs respectively. They are given by

$$\begin{array}{ccc} W_{S}^{+}\left(n_{S},n_{D}\right) & = & \text{Prob}(n_{S},n_{D}\to n_{S}+1,n_{D})\\ & = & (\frac{r_{2}\;\left(1-u_{2}\right)\;n_{S}+{\tilde{r}}_{2}\;\eta_{2}\;n_{D}}{N_{r}})\frac{N_{S}-n_{S}}{N_{S}},\\ W_{S}^{-}\left(n_{S},n_{D}\right) & = & \text{Prob}(n_{S},n_{D}\to n_{S}-1,n_{D})\\ & = & (\frac{r_{1}\;\left(1-u_{1}\right)\;\left(N_{S}-n_{S}\right)+{\tilde{r}}_{1}\;\eta_{1}\;\left(N_{D}-n_{D}\right)}{N_{r}})\;\frac{n_{S}}{N_{S}},\\ W_{D}^{+}\left(n_{S},n_{D}\right) & = & \text{Prob}(n_{S},n_{D}\to n_{S},n_{D}+1)\\ & = & (\frac{{\tilde{r}}_{2}\;\left(1-\eta_{2}\right)\;n_{D}+r_{2}\;u_{2}\;n_{S}}{N_{r}})\;\frac{N_{D}-n_{D}}{N_{D}},\\ W_{D}^{-}\left(n_{S},n_{D}\right) & = & \text{Prob}(n_{S},n_{D}\to n_{S},n_{D}-1)\\ & = & (\frac{{\tilde{r}}_{1}\;\left(1-\eta_{1}\right)\;\left(N_{D}-n_{D}\right)+r_{1}\;u_{1}\;\left(N_{S}-n_{S}\right)}{N_{r}})\;\frac{n_{D}}{N_{D}}. \end{array}$$ (2)

The denominator N_r_ denotes the total fitness of SC and DC individuals:

$$\begin{array}{c} N_{r}=r_{1}\;\left(N_{S}-n_{S}\right)+r_{2}\;n_{S}+{\tilde{r}}_{1}\left(N_{D}-n_{D}\right)+{\tilde{r}}_{2}\;n_{D}. \end{array}$$ (3)

The above Markov process has two absorbing states corresponding to fixation or extinction of the mutant or WT. The competition between the two genotypes in the stem cell compartment is tied to the competition inside the non-stem cell compartment via differentiation and dedifferentiation mechanisms. In the absence of plasticity we have a hierarchical population structure where only mutations in the stem cell compartments can give rise to fixation in the whole population.

In the next section, we present exactly calculated solutions for the probability of fixation of an invading mutant as a function of division rates and (de)differentiation rates. Our calculations match with simulation results for a wide range of parameters and population sizes.

### Fixation probability in a heterogeneous Moran process

One of the most important questions to address within a heterogeneous population is the chance of success for a mutation in different subtypes.

The fixation probability of a mutant originating in the stem cell compartment *ρ*_S_ or the non-stem cell compartment *ρ*_D_ is a measure of the tumor initiating capacity of each subpopulation. For a completely hierarchical population, only mutants that arise in the stem cell niche have a chance of fixating in the whole population thus *ρ*_S_ = *ρ*. If the progenitors can dedifferentiate into stem-like cells, the comparison between the two fixation probabilities, (*ρ*_S_ and *ρ*_D_), is a good measure of how the tumor initiating capacity correlates with the notion of stemness.

The use of the probability generating function (PGF) method to study a constant population Moran process is discussed in [58–60]. It is used to present an alternative derivation of the (well-mixed) Moran fixation probability, by identifying a martingale for the process. Here we generalize this technique for a heterogeneous population under selective pressure in the presence of phenotypic plasticity.

A martingale for the above four population model, Eqs (1) and (3), can be written as

$$\langle {\left(z_{\text{S}}^{⋆}\right)}^{n_{\text{S}}}{\left(z_{\text{D}}^{⋆}\right)}^{n_{\text{D}}}\rangle ,$$ (4)

where 〈⋅〉 denotes the stochastic average and the (auxiliary) variables $z_{S}^{⋆}$ and $z_{D}^{⋆}$ satisfy the following system of algebraic equations (see S1 File, Appendix A-C for more details on derivation of these equations)

$$\begin{array}{ccc} & & \left(z_{S}^{⋆}-1\right)[r_{2}\left(1-u_{2}\right)\;z_{S}^{⋆}-r_{1}\left(1-u_{1}\right)-{\tilde{r}}_{1}\;\eta_{1}]+\left(z_{D}^{⋆}-1\right)\;z_{S}^{⋆}\;r_{2}\;u_{2}=0\\ & & \left(z_{D}^{⋆}-1\right)[{\tilde{r}}_{2}\;\left(1-\eta_{2}\right)\;z_{D}^{⋆}-{\tilde{r}}_{1}\;\left(1-\eta_{1}\right)-r_{1}\;u_{1}]+\left(z_{S}^{⋆}-1\right)\;z_{D}^{⋆}\;{\tilde{r}}_{2}\;\eta_{2}=0. \end{array}$$ (5)

By matching the initial conditions for *t* = 0 and the steady state solutions of the PGF, we can obtain exactly calculated expressions for the fixation probability of mutants of each subtype (stem or non-stem). In general, the fixation probability beginning with *i* mutant SCs and *j* mutant progenitors is (See S1 File, Appendix A-C for details)

$$\begin{array}{c} \rho_{ij}=\frac{1-{\left(z_{\text{S}}^{⋆}\right)}^{i}{\left(z_{\text{D}}^{⋆}\right)}^{j}}{1-{\left(z_{\text{S}}^{⋆}\right)}^{N_{\text{S}}}{\left(z_{\text{D}}^{⋆}\right)}^{N_{\text{D}}}}. \end{array}$$ (6)

For *i* = 1 and *j* = 0 (starting with one initial SC mutant) the fixation probability is

$$\begin{array}{c} \rho_{\text{S}}\equiv \rho_{10}=\frac{1-z_{\text{S}}^{⋆}}{1-{\left(z_{\text{S}}^{⋆}\right)}^{N_{\text{S}}}{\left(z_{\text{D}}^{⋆}\right)}^{N_{\text{D}}}}. \end{array}$$ (7)

Similarly, the fixation probability of a newborn mutant in the DC compartment (*i* = 0, *j* = 1) is

$$\begin{array}{c} \rho_{\text{D}}\equiv \rho_{01}=\frac{1-z_{\text{D}}^{⋆}}{1-{\left(z_{\text{S}}^{⋆}\right)}^{N_{\text{S}}}{\left(z_{\text{D}}^{⋆}\right)}^{N_{\text{D}}}}. \end{array}$$ (8)

Moreover, assuming random mutations, under which the first mutation occurs randomly within the entire population to signify the same chance for the occurrence of the first mutant. Thus we consider uniform mutation rates for the both compartments, the average fixation probability is given by

$$\begin{array}{c} \rho =\frac{1-\left(N_{\text{S}}/N_{\text{tot}}\right)z_{\text{S}}^{⋆}-\left(N_{\text{D}}/N_{\text{tot}}\right)z_{\text{D}}^{⋆}}{1-{\left(z_{\text{S}}^{⋆}\right)}^{N_{\text{S}}}{\left(z_{\text{D}}^{⋆}\right)}^{N_{\text{D}}}}. \end{array}$$ (9)

with *N*_tot_ = *N*_S_ + *N*_D_. The probability of a successful emergent mutant *before* time *t* (from a background of *N*_tot_ normal cells) is given by

$$\begin{array}{c} P\left(t\right)=1-e^{-N_{\text{tot}}\cdot \mu \rho t}, \end{array}$$ (10)

where *μ* denotes the mutation rate.

### Stochastic simulation

Using the model described above, we performed numerical simulations using such updates until each of the runs tends to saturation in the fraction of SCs and DCs, or until we reach the maximum updating time of T = 15,000 for each realization. Then running the whole procedure for 20,000 realizations, we calculated the fraction of results for the fixation probability of SCs and DCs in those runs. Then repeating each calculation for a set of five iterations, we calculated the mean and error bars. Errors are calculated as the standard deviation of the mean.

### Replicator equation

The Markov process considered in our four—compartment model exhibits deterministic dynamics in the absence of stochastic fluctuations, i.e. infinite population limit. This replicator equation captures the average frequency of various phenotypes which provides insight into the evolutionary dynamics of the system. Firstly, we detect the frequency of each phenotype at the fixed points and secondly, analyze the phase diagram by varying different parameter values (see the Results Sec.) at equilibrium. Starting from the master Eq (1), one obtains the following system of deterministic equations for malignant SCs and DCs (see S1 File, Appendix D for derivation)

$$\begin{array}{ccc} \frac{dx_{S}}{dt} & = & \frac{\left[r_{2}\;\left(1-u_{2}\right)-r_{1}\;\left(1-u_{1}\right)\right]\;x_{S}\;\left(1-x_{S}\right)+{\tilde{r}}_{2}\;\eta_{2}\;x_{D}\;\left(1-x_{S}\right)-{\tilde{r}}_{1}\eta_{1}\;x_{S}\;\left(1-x_{D}\right)}{r_{1}\;\left(1-x_{S}\right)+r_{2}\;x_{S}+{\tilde{r}}_{1}\;\left(1-x_{D}\right)+{\tilde{r}}_{2}\;x_{D}},\\ \frac{dx_{D}}{dt} & = & \frac{\left[{\tilde{r}}_{2}\;\left(1-\eta_{2}\right)-{\tilde{r}}_{1}\;\left(1-\eta_{1}\right)\right]\;x_{D}\;\left(1-x_{D}\right)+r_{2}\;u_{2}\;x_{S}\;\left(1-x_{D}\right)-r_{1}\;u_{1}\;x_{D}\;\left(1-x_{S}\right)}{r_{1}\;\left(1-x_{S}\right)+r_{2}\;x_{S}+{\tilde{r}}_{1}\;\left(1-x_{D}\right)+{\tilde{r}}_{2}\;x_{D}}, \end{array}$$ (11)

where *x*_S_ = 〈*n*_S_(*t*)/*N*_S_〉 and *x*_D_ = 〈*n*_D_/*N*_D_〉 are the average frequencies of mutant SCs and DCs respectively. Stationary state frequencies, $x_{S}^{⋆}$ and $x_{D}^{⋆}$ satisfy the following coupled system of equations

$$\begin{array}{ccc} & & \;\left[r_{2}\;\left(1-u_{2}\right)-r_{1}\;\left(1-u_{1}\right)\right]\;x_{S}^{⋆}\;\left(1-x_{S}^{⋆}\right)+{\tilde{r}}_{2}\;\eta_{2}\;x_{D}^{⋆}\;\left(1-x_{S}^{⋆}\right)-{\tilde{r}}_{1}\eta_{1}\;x_{S}^{⋆}\;\left(1-x_{D}^{⋆}\right)=0,\\ & & \;\left[{\tilde{r}}_{2}\;\left(1-\eta_{2}\right)-{\tilde{r}}_{1}\;\left(1-\eta_{1}\right)\right]\;x_{D}^{⋆}\;\left(1-x_{D}^{⋆}\right)+r_{2}\;u_{2}\;x_{S}^{⋆}\;\left(1-x_{D}^{⋆}\right)-r_{1}\;u_{1}\;x_{D}^{⋆}\;\left(1-x_{S}^{⋆}\right)=0. \end{array}$$ (12)

In the next section, after obtaining the exactly calculated form of the fixation probability for this generalized Moran process, we will use Eq (12) to derive the condition for evolutionary advantage of the mutant genotype and the phase diagram for the model.

## Results

### Exactly calculated results for the fixation probability

We first begin with the exactly calculated expressions for the survival probabilities Eqs (6)–(9) where $z_{S}^{⋆}$ and $z_{D}^{⋆}$ are the solutions of Eq (5). We begin with some simpler limiting cases where compact algebraic results can be obtained, and then proceed to general solutions of Eqs (6)–(9). As we mentioned previously, for simplicity we have assumed that the death rates of normal and mutant (stem and non-stem) cells are equal. In general and when the death rates are not equal, to find $z_{S}^{⋆}$ and $z_{D}^{⋆}$, one can replace $r_{i},{\tilde{r}}_{j}$ $r_{i}/d_{i},{\tilde{r}}_{j}/{\tilde{r}}_{j}$ and the relative fitness *r* with *r*/*d* where *d* is the relative fitness of mutants when the death rates of normal individuals are normalized to 1.

#### Standard Moran process

Let us consider the simple case where the differentiation and dedifferentiation rates are set to zero. In this case our model should reduce to two disjoint Moran processes, one for the stem cell and one for the non-stem cell compartments.

For this case, we obtain $z_{S}^{⋆}=r_{1}/r_{2}$ and $z_{D}^{⋆}={\tilde{r}}_{1}/{\tilde{r}}_{2}$ and the fixation probability for a mutant to dominate a SC (DC) niche is

$$\begin{array}{c} \rho_{1}=\frac{1-\frac{r_{1}}{r_{2}}}{1-{\left(\frac{r_{1}}{r_{2}}\right)}^{N_{\text{S}}}},\;\rho_{2}=\frac{1-\frac{{\tilde{r}}_{1}}{{\tilde{r}}_{2}}}{1-{\left(\frac{{\tilde{r}}_{1}}{{\tilde{r}}_{2}}\right)}^{N_{\text{D}}}}. \end{array}$$ (13)

Another interesting limit that resembles a well-mixed Moran process in the stem cell compartment occurs when ${\tilde{r}}_{1,2}≃0$ but *u*_1,2_ ≠ 0. This is an exaggerated case showcasing the limited proliferation capacity of non-stem cell progeny. The corresponding average fixation probability for an emerged mutant in the SC or DC compartment is

$$\begin{array}{c} \rho =\frac{N_{\text{S}}\left(1-\frac{r_{1}}{r_{2}}\right)}{N_{\text{tot}}\left(1-{\left(\frac{r_{1}}{r_{2}}\right)}^{N_{\text{S}}}\right)}, \end{array}$$ (14)

since the fixation probability of a newborn mutant in the DC class will be zero in this case.

#### Invasion in hierarchical model (zero plasticity)

We now consider a more general case with no plastic potential, *η*_1,2_ = 0. For the moment, we assume $r_{1}={\tilde{r}}_{1}=1$, $r_{2}={\tilde{r}}_{2}=r$.

$$\begin{array}{c} \rho_{\text{S}}=\frac{1-\frac{1-u_{1}}{r\left(1-u_{2}\right)}}{1-{\left(\frac{1-u_{1}}{r\left(1-u_{2}\right)}\right)}^{N_{\text{S}}}},\;\rho_{\text{D}}=0,\;\rho =\frac{N_{\text{S}}}{N_{\text{tot}}}\rho_{\text{S}}. \end{array}$$ (15)

**Fig 2:**
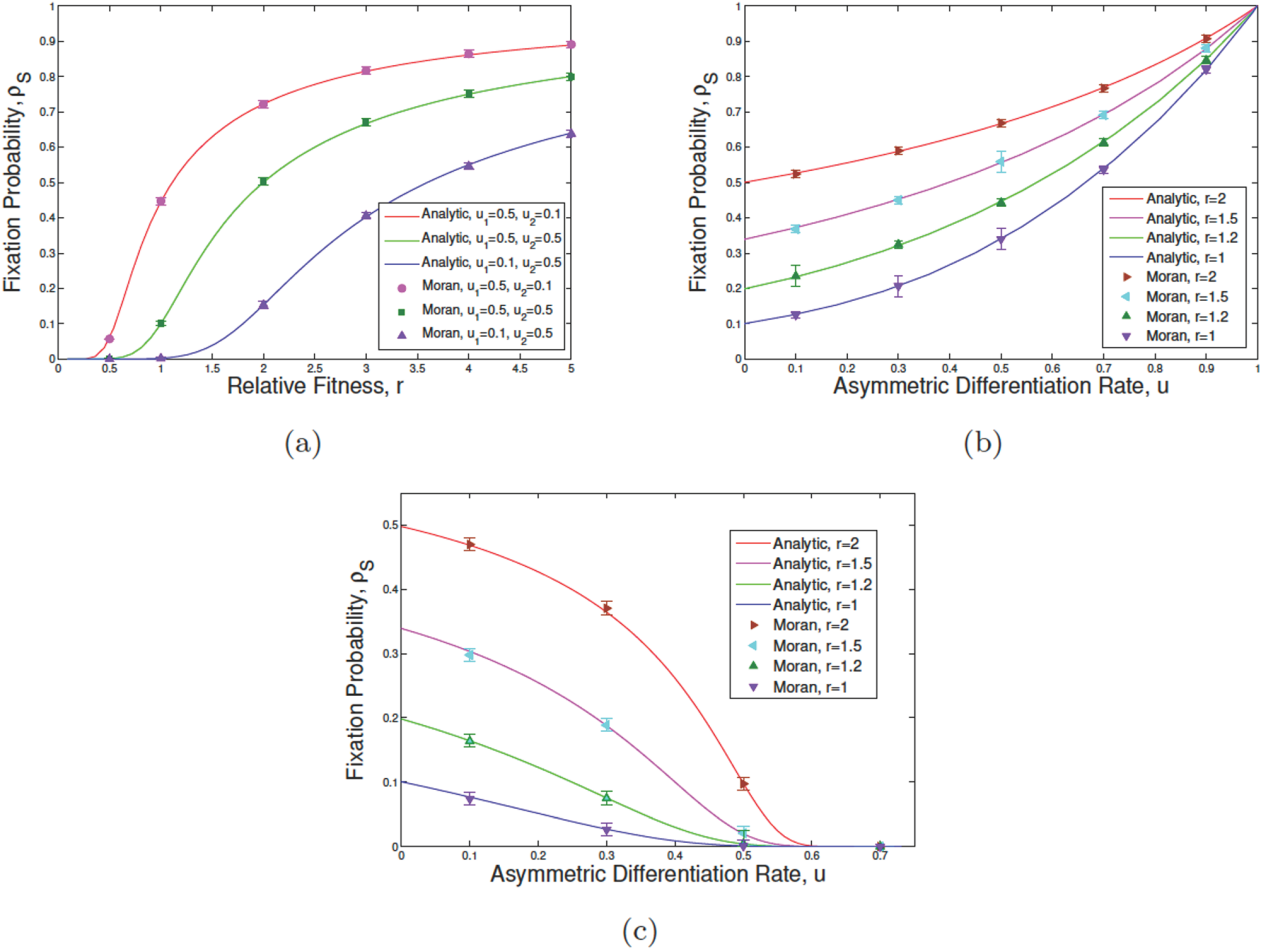
The fixation probability of mutants in the absence of plasticity. We assume *N*_S_ = *N*_D_ = 10, $r_{1}={\tilde{r}}_{1}=1$, and *η*_1_ = *η*_2_ = 0 in simulations (points) and exact calculations (solid lines) for an initial mutant in the SC compartment. Each error bar shown at each point is the standard error of the mean. In (a) changing parameters *u*_1_, *u*_2_, that are the differentiation rates of normal and tumor SCs respectively, the trends for the fixation probability of mutants is given as a function of relative fitness of mutants, referred to as $r_{2}={\tilde{r}}_{2}=r$. In (b) and (c) the fixation probability variation is given in terms of asymmetric differentiation rates *u*_1_ = *u*_2_ = *u* and various values of *r* and the ratio of the differentiation rates of normal SCs *ϵ* = *u*_2_/*u*_1_. In (b) *ϵ* = 0.5 and in (c) *ϵ* = 1.5.

Fig 2 shows how the survival chance of an initial mutant SC varies in terms of the relative fitness of mutant SCs/DCs as well as the probability of asymmetric division in the SC compartment. The population size is set to *N*_S_ = *N*_D_ = 10. The results are plotted for three sets of differentiation rates (*u*_1_ = *u*_2_ = 0.5), (*u*_1_ = 0.1, *u*_2_ = 0.5) and (*u*_1_ = 0.5, *u*_2_ = 0.1) as *r* varies (Fig 2(a)). If the normal cell differentiation rate *u*_1_ is decreased from the balanced limit of *u*_1_ = *u*_2_ = 1/2 the fixation probability decreases as well. Conversely, if the mutant cell differentiation rate, *u*_2_ is decreased, away from *u*_2_ = 1/2, *ρ*_S_ would increase.

We rewrite differentiation rates as *u*_1_ = *u* and *u*_2_ = *ϵu* where *u* stands for an overall measure of differentiation, in both genotypes, and *ϵ* measures the asymmetry in them. If *ϵ* < 1 the normal type differentiates more often per division and *ϵ* > 1 indicates that invader cells differentiate more often. Fig 2(b) shows *ρ*_S_ for *ϵ* = 1/2 as a function of *u* for several division rates, *r* = 1, 1.2, 1.5, 2. For high differentiation rates, *u*, the fixation probability converges to unity. This is consistent with Eq (15). The relative fitness of the mutant versus normal types are given by *r*(1 − *u*_2_)/(1 − *u*_1_) = *r*(2 − *u*)/(2(1 − *u*)). As *u* → 1 the relative fitness approaches infinity and thus the value of fixation probability tend to unity.

Similar results are plotted for *ϵ* = 3/2 in Fig 2(c). Now *ρ* approaches zero for large *u*. Again this can be seen since *r*(1 − *u*_2_)/(1 − *u*_1_) = *r*(2 − 3*u*)/(2(1 − *u*)) which approaches zero as *u* goes to 2/3.

#### Invasion in the presence of plasticity (dedifferentiation)

Now we consider a more general case with non-zero differentiation and dedifferentiation rates. As before we set $r_{1}={\tilde{r}}_{1}=1$. We parameterize the fitness of mutant subtypes as *r*_2_ = *αr* and ${\tilde{r}}_{2}=\beta r$. The parameter *r* denotes the overall proliferation advantage of mutants over normal cells.

**Fig 3:**
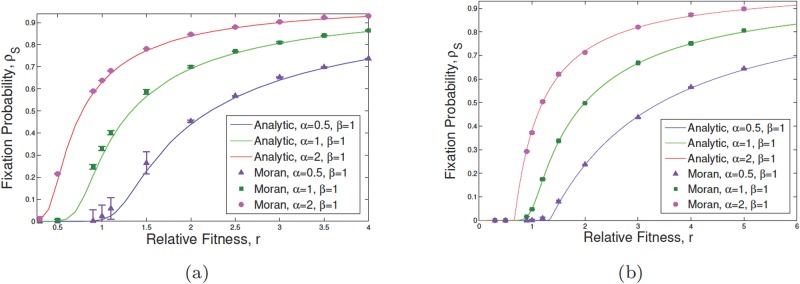
The fixation probability of mutants in the presence of phenotypic plasticity. We suppose that *N*_S_ = *N*_D_ = 10, $r_{1}={\tilde{r}}_{1}=1$, *r*_2_ = *αr*, and ${\tilde{r}}_{2}=\beta r$ in the exactly calculated results (solid curves) and stochastic simulation (points with bars as the standard error of the mean) starting with an initial mutant within SCs. Changing *α* from 0.5 to 1 and then to 2 while *β* remains fixed, the behavior of the system is shown in terms of the fixation probability with respect to the relative fitness *r*. In subfigure (a) plastic potential has only been considered for malignant individuals: *η*_1_ = 0, *η*_2_ = 0.5 while in (b) both WT and mutant cells can dedifferentiate to the stemness state (*η*_1_ = *η*_2_ = 0.5). Straightforward calculations reveal that for the given parameter values in (b), *ρ* = *ρ*_S_ = *ρ*_D_.

Fig 3 shows *ρ* as a function of *r* for various values of *α* and *β*. Fig 3(a) assumes plastic mutants only (*η*_1_ = 0, *η* = 0.5) whereas Fig 3(b) assumes equally plastic genotypes (*η*_1_ = *η*_2_). The differentiation rates are equal and set to 1/2 for simplicity. As can be seen, the overall increase in *r* increases the fixation probability monotonically in both cases. However, varying values of *α* and *β* leads to the neutral value for the proliferation potential *r*. For example, in the case of Fig 3(a) for *α* = *β* = 1, i.e. $r_{2}={\tilde{r}}_{2}=r$, the mutant is advantaged for *r* > 0.75. For *α* = 0.5, *β* = 1, however, *r* > 1.25 has *ρ* > 1/*N*. Interestingly if stem cells divide faster than progenitors, i.e. *α* > *β*, even for *r* ≈ 1/2 the fixation probability is larger than the neutral value. We used the neutral fixation probability as 1/*N*_S_ = 1/*N*_D_ = 1/10, which is the neutral *ρ* in the absence of (de)differentiation. Similar observations can be made in the case of *η*_1_ = *η*_2_ in part (b). As can be seen our results are in very good agreement with exact stochastic simulations.

**Fig 4:**
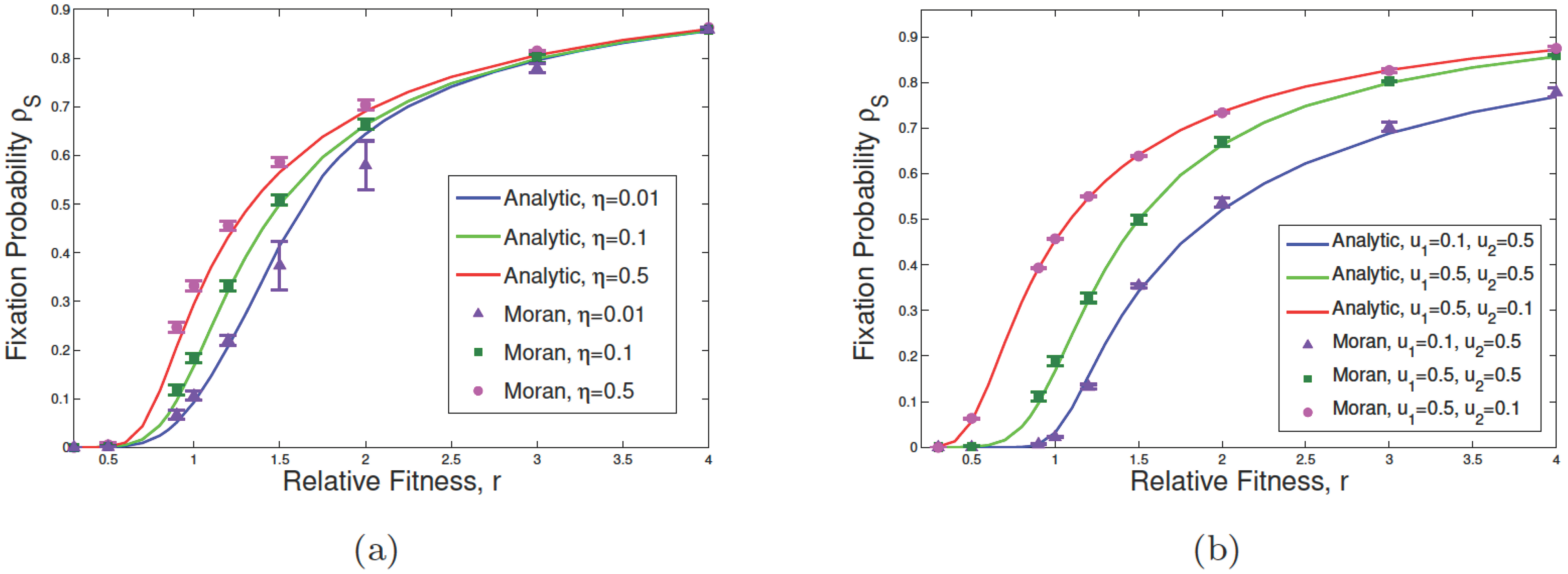
Effect of change in asymmetric differentiation and plasticity rates on survivability of mutants. Subfigures show the variation of *ρ*_S_ with respect to the relative fitness of mutants *r*, in which we start with an initial mutant within SCs. We assume that *N*_S_ = *N*_D_ = 10, $r_{1}={\tilde{r}}_{1}=1,$ and $r_{2}={\tilde{r}}_{2}=r$. In subfigure (a), the fixation probability of SCs as a function of *η* is given, where *η* = 0.01, 0.1, 0.5 while *u*_1_ = *u*_2_ = 0.5, and *η*_1_ = 0. In (b) *η*_1_ = 0 and *η* = 0.1. Changing parameters *u*_1_ and *u*_2_, which are the asymmetric division rates of normal and tumor SCs respectively, the fixation probability as a function of *u*_1_, *u*_2_ is shown. Solid lines represent the exact calculation and points correspond to simulation results (error bars are based on the standard error of the mean).

In Fig 3, *ρ*_S_ is plotted as a function of *r* for several values of *u*_1_ and *u*_2_ (part (a)) as well as *η* (part (b)). The effect of dedifferentiation on increasing the value of the fixation probability is most significant for *r* values corresponding to the neutral limit (Fig 3(b)) and Fig 2(a). For example in Fig 3(b) value of *ρ* near *r* = 1 increases from approximately 0.1(≈ 1/*N*) to 0.3 as *η* increases to 0.5 (from 0.01). However for *r* = 4 the difference between the two values of *ρ* for *η* = 0.01, 0.5 is negligibly small. Moreover, Fig 4 represents the effect of variation in asymmetric differentiation rates, *u*_1_ and *u*_2_, as well as the phenotypic plasticity rate *η* of malignant DCs. Particularly, the change in asymmetric differentiation of normal cells shows more effect on the fixation probability of SCs in comparison to that of malignant cells.

**Fig 5:**
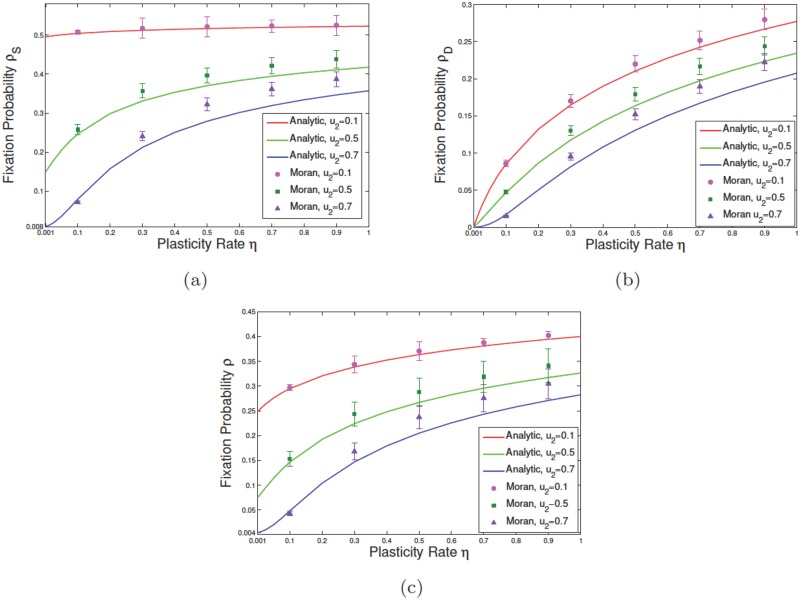
Effect of change in the rate of phenotypic plasticity for mutant DCs on the survival probability of malignant cells. In subfigures (a), (b), and (c) we have respectively considered the fixation probability of mutants while the initial malignant mutation respectively occurs in SC, DC, and SC+DC compartments at random. In these plots, we assumed that *N*_S_ = *N*_D_ = 10, $r_{1}={\tilde{r}}_{1}=1$, $r_{2}={\tilde{r}}_{2}=1.1,u_{1}=0.5$ and *η*_1_ = 0. Different asymmetric division rates of normal and malignant individuals have also taken into account when the fixation probability is given as a function of *η*. Solid curves and points are respectively the results of exact calculations and simulation analyses. At each given discrete point, the error bar depicts the standard error of the mean.

In the presence of dedifferentiation, mutations arising in the differentiated compartment can now undergo clonal expansion. In Fig 5 we plotted values of the fixation probabilities *ρ*_S_, *ρ*_D_ and the average fixation *ρ* as a function of *η*. As expected for *η* = 0, *ρ*_D_ tends to zero for various values of *u*_1_ and *u*_2_. However *ρ*_S_ approaches the results obtained before (Eq (15)). In Fig 5(a) for *u*_1_ = *u*_2_ = 0.5 and *r* = 1.1, *ρ*_S_ approaches 0.15 as *η* = 0. This is in agreement with the extended Moran result, Eq (15), with effective fitness *r*(1 − *u*_2_)/(1 − *u*_1_) which for *N*_S_ = 10 gives *ρ*_S_ = 0.15. As *η* increases *ρ*_S_ increases further and approaches 0.4 for large values of *η*. We can see that finite values of *η* have now conferred a selection advantage on the previously deleterious mutants. For example for *u*_1_ = 0.5, *u*_2_ = 0.1 and *r* = 1.1, the effective fitness is less than unity with *ρ*_S_ ≈ 0. For finite values of the dedifferentiation rate, *ρ*_S_ will exceed the neutral value 1/*N*. For example for *η* = 0.5 we can read *ρ*_S_ ≈ 0.25 in this case.

In Fig 5, by increasing *u*_2_, for higher values of plasticity, exact calculations and numerical simulation show a slightly increasing difference, which may arise as a result of a high-level competition between asymmetric differentiation of mutant SCs and dedifferentiation of mutant DCs which results in a slight deviation of the stochastic simulations from the exactly calculated curves. In reality the rate of dedifferentiation is not high in some epithelial layers (for instance see [48]) and in this case, exactly calculated and simulation results are in agreement.

The exact calculations and stochastic simulations, both declare the fact that polarity in division may play a more important role in the destiny of malignancy than phenotypic plasticity. The results of this section, which cover the all possible scenarios for the occurrence of the first one-hit mutant within whole population, shows how the evolution of mutants would change a normal population to an abnormal population. Moreover, based on our analysis one can conclude that dedifferentiation can only affect a nearly neutral system. Furthermore, in a system with higher relative fitness of mutants, the asymmetric division rate of normal SCs reveals a higher impact on the survivability of mutants rather than the asymmetric division rate of mutant SCs.

In the next section, finding the replicator dynamics of mutant SCs and DCs, we find a phase diagram for change in asymmetric division rate and dedifferentiation and show how the phase digraph explains the high fixation probability of disadvantageous mutants.

### Phase diagram

To derive the phase diagram that determines the evolutionary advantage of a malignant genotype, we begin with the system of Eq (11). The fixed points of the replicator equation determine the stationary frequencies of mutant subtypes: $x_{S}^{⋆},x_{D}^{⋆}$. There are four fixed—points for the system of Eq (11). The fixed point $\left(x_{S}^{⋆}=0,x_{D}^{⋆}=0\right)$ corresponds to the extinction of both phenotypes and other fixed points $\left(x_{S}^{⋆}=1,x_{D}^{⋆}=1\right)$ or any $\left(x_{S}^{⋆}\neq 0,x_{D}^{⋆}\neq 0\right)$ corresponds to successful invasion by the mutant. We do not distinguish between these fixed points as the stochastic fluctuations prohibit coexistence of mutant and WT phenotypes. We determine the phase boundary when a fixed point $\left(x_{S}^{⋆},x_{D}^{⋆}\right)$ merges with (0, 0). This indicates that the invasion has become unstable to parameter changes, and determines the phase boundary in the phase space of parameters.

Similar results can be obtained from the Eqs (6)–(8) requiring that *ρ*_S_ = 1/*N*_S_ (corresponding to the neutral selection). In a large population, this approach would be identical to the results from the replicator equation.

At first, we assume *u*_1_ = *u*_2_ = *η*_1_ = *η*_2_ = 0, then the solution to the system (12) results in two fixed points, $\left(x_{S}^{⋆}=0,x_{D}^{⋆}=0\right)$ (extinction) or $\left(x_{S}^{⋆}=1,x_{D}^{⋆}=1\right)$ (fixation) of invading mutant SCs (or DCs).

Next, we consider the case with $r_{1}={\tilde{r}}_{1}=1$, $r_{2}={\tilde{r}}_{2}=r$, *u*_1_ = *u*_2_ = *u*, *η*_1_ = 0, and *η*_2_ = *η*. We have plotted the phase diagram for the model parameter space of *η* − *u* − *r*. The phase boundary that determines the condition for neutrality is given by the algebraic relation (see S1 File, Appendix D for details)

$$\begin{array}{c} \left(u+\eta -1\right)r^{2}+[-u^{2}+\left(\eta -1\right)u+2-\eta ]r-1+u^{2}=0, \end{array}$$ (16)

**Fig 6:**
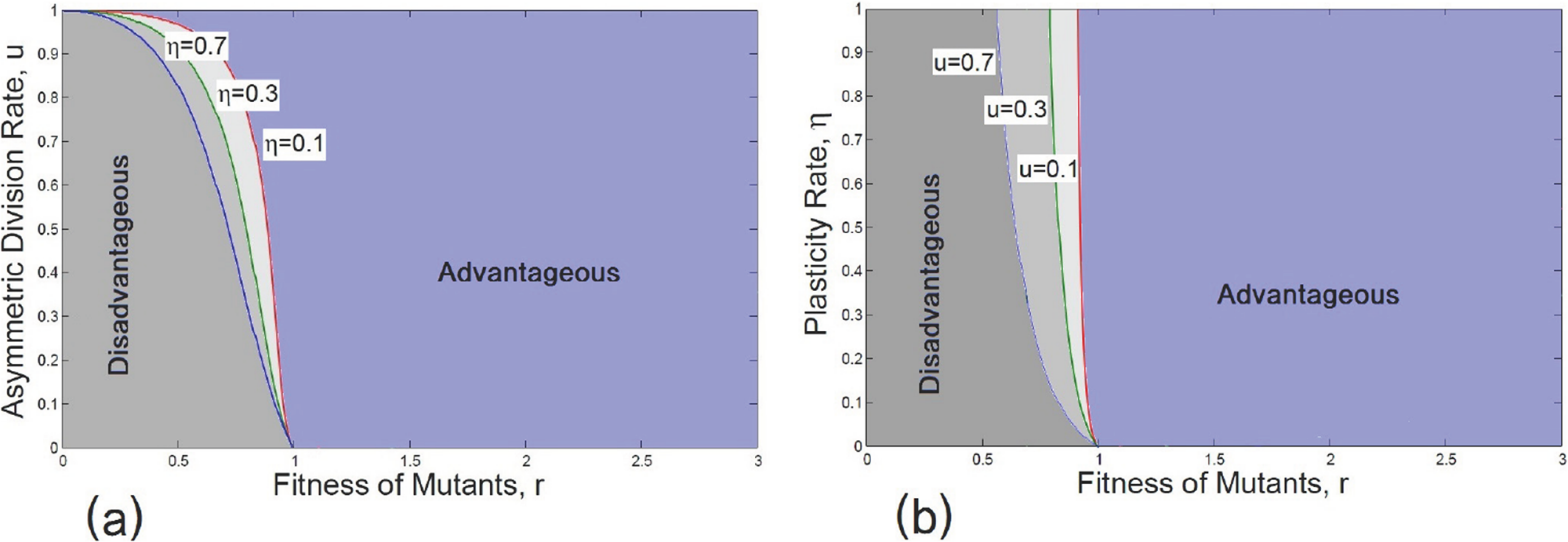
Phase diagram of plastic mutant SCs. The phase boundary for advantageous and disadvantageous mutant populations are given as differentiation and plasticity rates change. We assume that $r_{1}={\tilde{r}}_{1}=1$, $r_{2}={\tilde{r}}_{2}=r$, *u*_1_ = *u*_2_ = *u*, and *η*_1_ = 0. Different regions for advantageous and disadvantageous mutant SCs are given in (a) as *u* changes. A similar analysis has been carried out in (b) as *η* varies. In (a) *η* = 0.1, 0.3, 0.7, here the alteration in the plasticity rate of DCs results in a tendency to approach various regions of fixation for mutant SCs, while the extinction domain shrinks with increasing *η*. In (b) *u* = 0.1, 0.3, 0.7. Increasing the asymmetric division rate *u*, the region for advantageous mutants expands to provide a higher survival chance for mutant SCs. In both cases, advantageous criteria relate to either fixation of mutants or coexistence of mutants and WT individuals.

The phase diagram is plotted in the space of *u* − *r* (Fig 6(a)) and *η* − *r* (Fig 6(b)). The observation that an increase in *η* can turn a previously deleterious mutant into a beneficial one, can be seen here as well. For example in Fig 6(b) for *u* = 0.7 and *r* = 0.9 < 1 where we expect a deleterious mutant for large enough values of *η*, we can cross the phase boundary into an advantaged region. Interestingly we observe a similar trend as the overall differentiation rate increases.

The exact calculations and stochastic simulations, both confirm the fact that polarity in division may play a more important role in the destiny of malignancy than phenotypic plasticity. The results of this section, which cover all the possible scenarios for the occurrence of the first one-hit mutant within the whole population, show how the evolution of mutants would change a normal population to an abnormal population. Moreover, based on our analysis, one can conclude that dedifferentiation can only affect a nearly neutral system. Furthermore, in a system with higher relative fitness of mutants, the asymmetric division rate of normal SCs reveals a higher impact on the survivability of mutants as opposed to the asymmetric division rate of mutant SCs. In the next section, finding the replicator dynamics of mutant SCs and DCs, we find a phase diagram for change in asymmetric division rate and dedifferentiation and show how the phase diagram explains the high fixation probability of disadvantageous mutants.

Regarding the above explanation, the effect of dedifferentiation can be seen in the context of cancer, where through the epithelial-mesenchymal transition (EMT), non-stem cell progenitors start to circulate in the body and find a new niche in which to grow [6]. These type of cells do not have as a high proliferation rate as stem cells but can undergo phenotypic plasticity. Based on our results in this section and according to Fig 6(a) and 6(b), we can conclude that although these type of cells are not advantageous, they do have a higher potential for dedifferentiation [61] in distant tissue, and become advantageous by switching back to the stemness state. Therefore, our multi-compartmental study may have the potential to capture the EMT mechanism of progenitor cells, known to drive the aggressive side of many cancers [6].

### Application to colorectal cancer

Clonal expansion in colorectal cancer is known to be initiated as a result of mutations occurring at the bottom of the stem cell niche [62]. More recently, Vermeulen *et al* [18] considered the dynamics of cells at the bottom of a normal colonic/intestinal crypt. Due to the structure of stem cells at the bottom of the crypt, the authors investigated the fate of an imposed mutant within functional stem cells and repeated this experiment for several oncogenes and tumor suppressor genes. One can consider the structure of stem cells in the niche to be a circular model of 4–8 functional stem cells [18]. The fixation probability of a single mutation and the relative fitness r of the mutant cells, compared with the normal host cells, are reported in [18] within such a circular structure. These estimates reveal that the relative fitness of the original cell containing APC^−/−^ is *r* = 1.58, while is *r* = 1.56 for Kras^*G*12*D*^, and *r* = 0.96 for P53^*R*172*H*^ (compared with normal control cells in mice). P53 mutation seems not to confer a fitness advantage and is weakly deleterious. However, for P53^*R*172*H*^, it has been suggested that the fitness of a mutant elevates from 0.96 to 1.16 in comparison with the DSS-treated cells (colitis) under inflammatory environment. Thus, having inflammatory signaling effects, has resulted in a selection advantage and thus a higher fitness for the P53^*R*172*H*^ mutants [18, 63].

**Fig 7:**
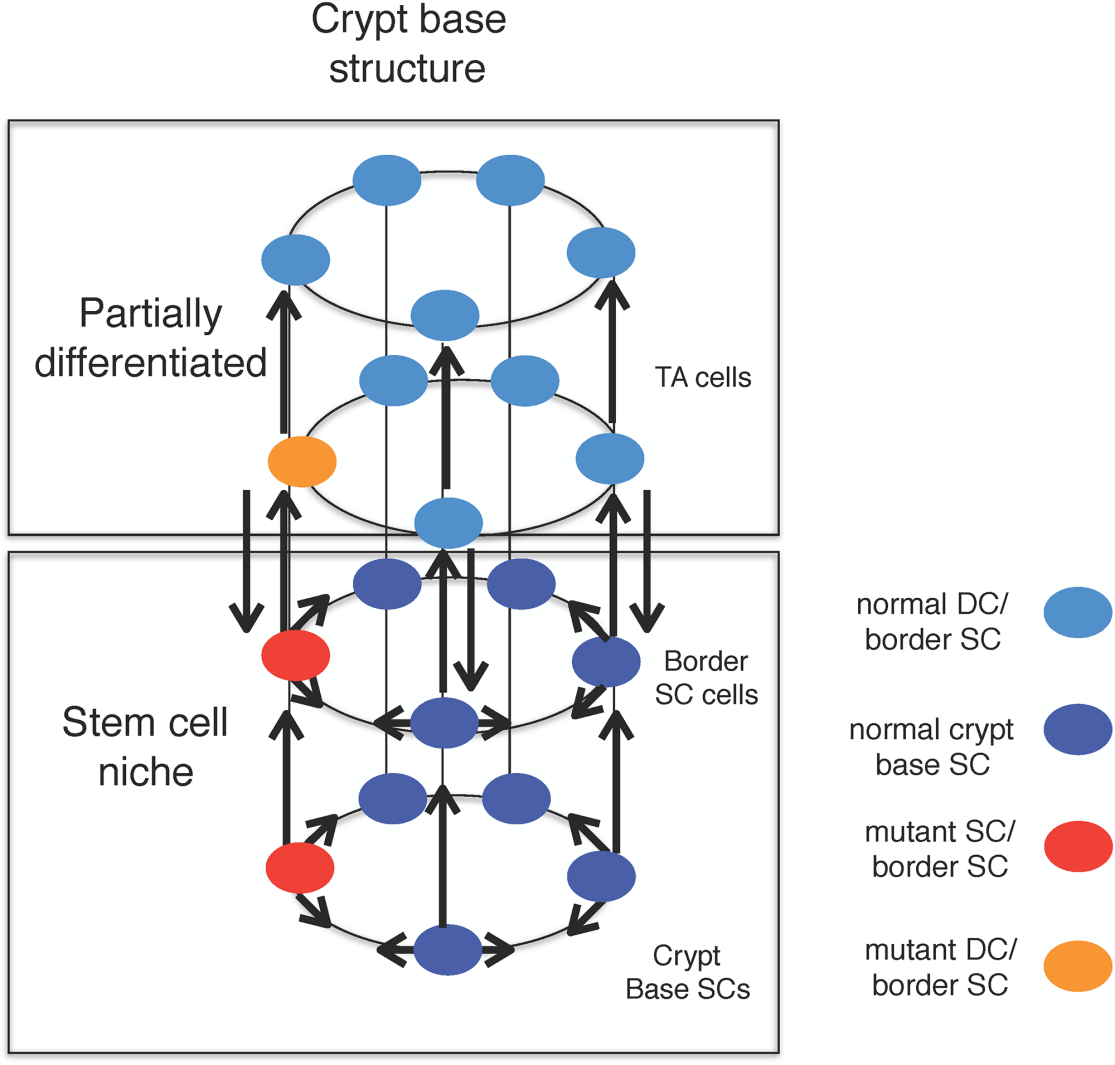
Cellular interactions in the colonic crypt as a newborn mutant arises within the stem or non-stem cell compartments. Within this schematic cylindrical model, we represent how our model is structured through the four compartments of host and mutant stem and non-stem cells. In contrast to the circular model of five SCs considered in [63], we assume a cylindrical model of two circles, one on the top of the other. SCs are located at the bottom circle while the circle on the top is full of partially DCs.

To stochastically investigate this mechanism, we consider a cylindrical model for the crypt-base, where stem cells are located in a compartment. One may consider a spatial structure for the stem cells, but for sake of simplicity, we assume that their location can vary within the compartment in our analysis (see Fig 7). At the top of this compartment, transit amplifying cells are positioned in another compartment in the colonic/intestinal crypt. More mature (non-stem) cell are also placed in higher layers. The bottom compartment of stem cells, in fact, consists of the central SCs and border SCs, while DCS (non-SCs) reside on the other subsequent upper layers. To compare the result of our model with the experimental observation in [18], for the fixation probability of P53 DSS colitis, we consider two compartments of SCs and DCs (see Fig 7). Thus, we have the number of stem cells (NS) approximately around 10 (see e.g. [10]) where only a fraction of these cells is functional. Fig 7 represents a hierarchy of cells at the bottom of the crypt. Moreover, it reveals how the progeny of an imposed mutant may develop among diverse layers.

Due to micro-environmental effects, a plastic DC in an upper layer may dedifferentiate to lower layers and become a stem-like cell. Fig 7 also shows how our model is connected to the recent experimental data [10]. Not only may the central stem cells at the bottom of the crypt be functional, but also the border stem cells (in the higher layer(s)) can be functional [10]. Hence, one may consider the functional cells together within the entire stem cell niche in one compartment. The reported fitness for cells with P53 mutation in [18] has been only calculated considering the functional SCs at the niche of the crypt, which coincides with our model. Therefore, putting both subpopulations of stem and border SCs together, one may assume that there exists approximately 10 functional SCs in the mouse crypt. Thus, considering a population of partial DCs on the top of this compartment (with ND = 10), we calculate the fixation probability of mutants in such a system.

Now, due to the similarity between human and mouse crypts in terms of the multi-compartmental structure of stem and non-stem cells, as well as due to the diverse proliferation rates in different compartments [18, 64–66], we may infer the same sort of results for the human crypt. A mouse crypt is comprised of 5–7 functional stem cells [18, 67] while the number of central and border SCs is estimated to be 8–16 in human [55].

On the other hand, according to the recent *in vivo* study by Schwitalla *et al*. [68], inflammatory signaling plays a role in elevating the rate of dedifferentiation. It has been also shown that inflammatory disease activates the transcription factor NF-*κ*B. NF-*κ*B which can, in turn, elevate Wnt-signaling which leads to the phenotypic plasticity of non-SCs [69]. Thus, our model suggests that the higher survival chance of the P53 mutated SCs along with the DSS-treated cells, may be the result of dedifferentiation in the presence of inflammatory stroma [68]. The survival probability of mutants in the colon/intestine, in the presence of inflammatory signaling, is presumably correlated with both the fitness and plastic nature of the epithelial cells. Thus, the fixation probability reported in [18] of cells with P53 DSS colitis mutation, can be derived using the same fitness *r* = 0.96 for P53 when dedifferentiation occurs.

Knowing more about the dynamics of colorectal cancer (as our research suggests) would help to identify more effective therapies such as cell transplantation to cure malignancies arising in the crypt. More precisely, our model suggests that replacing the niche of the crypt with healthy stem cells via transplantation, would result in removal of the cancer cells in the rest of the crypt (see Fig 5(b) in which *ρ*_D_ is negligible for smaller dedifferentiation rates.

**Fig 8:**
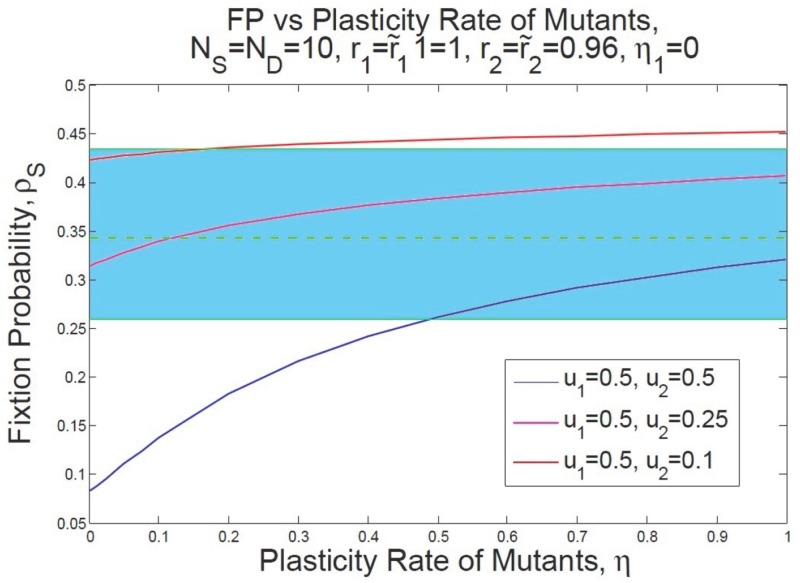
Expectations for the enhance in the fixation probability of P53 mutants under inflammation. We propose that when microenvironmental-induced plasticity can occur as a result of inflammatory injury, the increase in the survival chance of mutants reported in [18] may occur as a result of dedifferentiation rather than an enhance in the fitness of P53 mutants. The blue strand show the range of change for the fixation probability represented in [18] with an average shown by dashed green line and error bars shown by green lines. We assume that $N_{S}=N_{D}=10,r_{1}={\tilde{r}}_{1}=1,r_{2}={\tilde{r}}_{2}=0.96,$ and *η*_1_ = 0. The blue, pink, and red lines are drawn for diverse values for the asymmetric differentiation and fitness of normal SC and non-stem cells are normalized to one while that of mutant SCs/non-SCs are equal to the reproduction rate reported in [18]. Then the fixation probability *ρ*_S_ is given over a range of different plasticity rates. When *u*_1_ = 0.5, *u*_2_ = 0.25, and *η* = 0.12 then *ρ*_S_ = 0.343 as has been reported in [18] with an increases in the reproduction rate of mutants from 0.96 to 1.16.

In Fig 8 our hypothesis about the effect of phenotypic plasticity on the fixation probability of mutants is illustrated. In this figure we show how an increase in the fixation probability of P53 mutants under inflammatory effect is possible for a range of different dedifferentiation rates. The dashed green line denotes the average value for the fixation probability of P53 mutants under inflammatory conditions and the blue region represents the error bar of this average value as reported in [18].

For instance, when *r* = 0.96 and *u*_1_ = 0.5, *u*_2_ = 0.25, having the plasticity rate at *η* = 0.12, one obtains the same fixation probability as [18] (see Table 2). This finding suggests that there is an elevation in the fixation of a deleterious mutant into an advantageous trait due to the plastic properties of the mutant (see also Discussion).

**Table 2:** Comparison between our exactly calculated result and experiment [18].

	*r*	*η*_2_	*ρ*_S_	Reference
P53 Controlled	0.96	0	0.113	[18]
P53 DSS Colitis	1.16	0	0.343	[18]
P53 DSS Colitis+Plasticity	0.96	0.12	0.343	Results Section

## Other applications: From AML pathogenesis to adaptive drug-resistance mechanism

Our findings can be analyzed not only in the study of colorectal cancer, but also to understand various mechanisms in different biological systems. The crucial component in our investigation, dedifferentiation, can be specifically demonstrated for diverse ecosystems [6, 19, 24–26]. We argue that our model may provide a better understanding of such mechanisms. Other potential applications of our model (in the presence of dedifferentiation) arise in diverse disciplines.

In the context of cancer, a set of human mammary epithelial cells have been reported that spontaneously switched to SLCs [70]. Moreover, for different purified populations of CD44- CD24+ non-CSCs, a shift to CD44+ CD24- CSCs *in vivo* has been reported in [70]. Furthermore, in breast cancer epithelial-mesenchymal transition (EMT) factors have been implicated in the production of SLCs from non-SCs [7, 22]. Gupta *et al* [23] have also observed that the epithelial DCs with Basal markers can convert to cells with SLC markers (see also [7] for more explanation). The authors were able to capture this behavior through a simple model of phenotype switching between stem-like state and differentiated state (see [40, 71]). Thus, after determining the required parameters experimentally, it may be possible to predict some of the features of the behavior of the system in terms of the fate of mutants.

Another potential application of our model is associated with an invasive type of leukemia, acute myeloid leukemia (AML). The dynamics of such a complex mechanism has still remained unclear even at steady state or after the reconstitution of the blood [72]. In the blood system, high levels of cell turnover occurs in the reproduction rate of approximately 10^12^ cells per day. For this purpose, progenitor cells which arise from hematopoietic stem cells (HSCs) generate the hierarchy of functionally mature hematopoietic cells. This hierarchy is initiated by HSCs and leads to multi—potential SCs, then to lymphoid/myeloid progenitors and finally to white cells, red cells, and platelets. Moreover, there is supporting experimental evidence for the occurrence of dedifferentiation in acute myeloid leukemia (AML). In these observations, the frequency (population fraction) of those non-SCs with the phenotype switching potential has been found to be lower than that of the original CD34+ CD38- leukemia-initiating cells [70, 73]. This suggests that there is a higher chance for more mature leukemic cells to experience phenotypic plasticity. Moreover, it is becoming more and more apparent that cellular dedifferentiation is activated in cells within a number of organs for tissue regeneration [2, 16, 74, 75].

To understand the role of different types of cell division and dedifferentiation in AML, one may simplify this hierarchy and only consider two different phenotypes: HSC and non-HSC compartments. Then our analysis could help clarify how this mechanism might favor or suppress the development of leukemia in hematopoietic systems. Unfortunately there is a lack of a suitable database from which to determine the diverse rates for reproduction, elimination, polarity, and dedifferentiation in normal and mutant hematopoietic cells. Thus it is essential to quantify these rates through specific experiments to measure these rates for both SCs vs. non-SCs.

Our model suggests that appropriate treatments should be applied based on the selection pressure exerted on the system either those treatments which control asymmetric differentiation, plasticity, or those that exchange the CSCs through SC transplantation, to prevent the growth of AML.

Alternatively, phenotypic plasticity may also play a role in the development of adaptive or non-adaptive traits in response to environmental pressures. The results of this study have implications for the evolution of adaptive resistance in tumor cells. Adaptive resistance is a specific phenotype associated with the resistance of a small population of cells under the effects of chemotherapy or radiation [76–78]. The adaptability of this phenotype provides a higher growth rate of this small population after therapy [79–81]. Based on the co-operation among SCs and DSc in each of the normal and cancer genotypes, even if all the mutant SCs are removed by therapeutic treatment, mutant cells will survive provided resistant DCs still exist. More precisely, the adaptively resistant DCs may switch back to a stemness state and thus may undergo relapse. Therefore, our model illustrates that the remission process after treatment, depends not only on the resistant SCs, but also on the adaptive resistant DCs. This emphasizes the fact that there is a crucial need for an intense combination therapy to control both adaptive resistance stem and non-stem cells.

We believe that plasticity in a hierarchical heterogeneous system is a crucial concept and has important implications, not only in cancer evolution but also in evolutionary biology, ecology, population genetics, and physiology [48, 53, 82–86]. The interplay between phenotypic plasticity and selection pressure in a heterogeneous population (under fluctuations in environmental conditions) has remained one of the open questions in the field [87–89]. On the other hand, since there are currently no accessible methods to control and fine-tune environmental effects, a theoretical study may throw light on the impact of various types of division as well as on the role of plasticity in natural selection of beneficial, deleterious, or neutral mutations.

## Discussion

The evolutionary implications of epigenetic heterogeneity are not very well understood in cancer biology. A known picture for phenotypic heterogeneity (when the genotypes are assumed to be identical) relates to the cancer stem cell hierarchy. In this picture, pluripotent cells with tumor initiating capacity can undergo mitotic events and either replenish their own population or produce a lineage of partially DCs (including precursor and/or transit amplifying cells).

In the current study, we present a general model of four distinct subpopulations to investigate Darwinian evolution in such a hierarchical structure. We consider two genotypically different populations (mutant and wild-type). Mutations are results of unwanted oncogenic or TSG mutations. Each genotype has phenotypically different subtypes of stem cells (SCs) and non-stem cells (DCs). SCs ultimately generate their associated progenies, DCs, through proliferation and asymmetric differentiation. DCs have restricted proliferation capacity. Due to the tissue structure of the crypt, the population of different subtypes remains approximately unchanged. Genetic mutations can occur among SCs or DCs which we assume to be occurring through a uniform probability. Mutations can confer not only higher division rates but also different rates of differentiation and plasticity among mutant subtypes. These changes can be triggered, for example, by microenvironmental conditions.

Our model predicts the fixation probability of a newborn mutant as a function of the division rates of mutant and resident SCs/DCs, differentiation and dedifferentiation rates. Exact calculation and numerical simulations—which are in almost perfect agreement—suggest that the asymmetric differentiation in the SC group has a major effect on the fate of mutants compared with dedifferentiation. More specifically, a greater impact on the fixation probability of SCs can be observed by the change in asymmetric differentiation of normal cells compared with that of malignant cells. Furthermore, we observe that the more plastic trait has an evolutionary advantage. This is most notable close to the neutral limit, i.e. when the proliferation rates of the mutants and residents are very close in value. Most interestingly, a sufficient increase in the rate of plasticity can turn a previously deleterious mutant into a beneficial one.

As an important application of this model, we consider the intestinal/colonic crypt with two groups of SCs and their neighboring, partially DCs. The competition between malignant mutations and normal cells in the base of the crypt has attracted much research interest, and is one the most studied scenarios in cancer evolution. It has recently been shown that the Moran type process, initially suggested by [60, 85, 90–93] are in perfect agreement with the experimental observation [63]. In this *in vivo* experimental analysis, *KRAS*, *APC*^+/−^, *APC*^−/−^, and *P*53 DSS colitis mutations separately induced in the crypt base. Assessing clonal lineage tracing [63], the authors were able to observe the growth of mutants in the populations and thus measured the fixation probability of mutants. However, the experimental observations sometimes ignore the microenvironmental interaction between the neighboring transit amplifying (TA) cells and SCs in the crypt (See [10, 63] for the models and estimated parameter values of population dynamics of the crypt, as well as numbers of central and border SCs at the base of the crypt).

In this investigation, we have suggested a plausible estimation method for the dedifferentiation rate which can give the same value of the fixation probabilities of P53 inactivation in the crypt base as observed by [18]. As the authors in [18] noticed, P53 mutations can maintain advantageous features to invade the crypt base in the presence of inflammatory signaling. We propose that this could potentially be a result of dedifferentiation caused by an inflammatory stroma [68]. This finding supports our idea about the elevation of a deleterious mutant into one with an advantageous trait due to the plastic properties of the mutant.

Kaveh *et al* [49] define a three compartment model comprising normal and mutant SC compartments and one further DC compartment which may contain both normal and mutant DCs, where different types of cell divisions and dedifferentiation of plastic DCs (in a homeostatic population of fixed size) are taken into account. Then by finding the replicator dynamics of normal and mutant SCs, the authors find numerical solutions to the replicator equations which determine the frequencies of diverse types of cells and the time to fixation of mutants in this system.

Moreover, Jilkine *et al* [48] consider a hierarchical structure of stem and progenitor cells where non-SCs may produce stem-like cells through dedifferentiation. Then using a semi-exact approximation, the authors estimated the time to fixation and showed that dedifferentiation enhances the selection pressure of SCs when the population size remains unchanged and results in exponential growth of a population with a stochastically altering population size and when the reproductions rates are frequency dependent. Furthermore, in [53], the authors discuss the rate of evolution in a simple hierarchical stem and non-stem cell population, without studying further compartments. They argue that stem cell symmetric division is preferred under natural selection for two-hit mutations, where symmetric division of SCs in comparison to asymmetric division would increase the rate of producing a first double-hit mutant. These authors have not considered the role of plasticity in such a system.

The impact of migration on the role of central and border stem cells has been also studied in [54] where only a bi-compartmental structure of the stem cell niche has been used. Furthermore, Mahdipour *et al* [55] have considered a general multi-compartmental structure for the colonic/intestinal crypt to analyze the fixation probability and time to fixation in the crypt. The authors have also investigated the effect of migration between the central and border SC compartments as well as the effect of the presence of immortal cells inside the colonic/intestinal crypt but without studying the effect of dedifferentiation on the fate of mutants. However, the current work presents an exact calculation of the fixation probability of a mutant (wherever the location of the initial mutant is) for these models where a phenotypic hierarchy of individuals among with polarity in division and dedifferentiation are considered (where each subpopulation of SCs and DCs has a constant population size).

We provide an exact approach to calculate the fixation probability of mutant for a 4-compartment structure, for the first time. By imposing the first mutant (one-hit mutant) at different locations within the entire population we found that there exists a cooperation among normal and also mutant subclones which guarantees the existence of both SCs and DCs groups in each of these subclones. Then comparing the role of asymmetric division vs. phenotypic plasticity, we find that asymmetric division may have a more pronounced effect on the progression of cancer. However, plasticity enhances the survival chance of disadvantageous mutants under certain environmental situations.

To elaborate further, our analysis also suggests some therapeutic outcomes. Firstly, we note the similar structure and dynamics of the crypt in mice and humans, and consider two stem cell populations of central and border stem cells in the human crypt like those in the mouse crypt [18, 63–65]. Our findings predict that SC transplantation may be a potential treatment which can eliminate malignancy from the rest of the crypt with high probability, provided the dedifferentiation rate is limited. In this case, stem cell transplantation would tend to eliminate the malignancy. When dedifferentiation occurs at a high rate, this type of therapy could be effective when the CSCs have a high fitness due to the selection pressure of the system. Moreover, we describe how our findings might be of assistance in understanding the adaptive drug-resistance mechanism, where a small portion of resistance DCs may lead to relapse even if all the cancer stem cells are eliminated by chemotherapy or radiation. Another application of our research could be in developing a better understanding of the EMT mechanism of progenitor cells and how dedifferentiation of these types of cells explains the initiation of metastatic cancers after EMT, even though the migrated cells have a lower proliferation rates.

This research is one of many mathematical approaches recently used to investigate various aspects of tumorigenesis. In our approach, we attempt to dissect a part of the complex machinery in multi—compartmental models, and understand this in greater detail. This study provides various insights concerning some features of initiation and progression of cancer, and suggests possible experimental investigations to confirm some of the theoretical results. Despite the generality and novelty of the present study, the derived results and conclusions still need to be validated through carefully designed experiments in the different disciplines where we seek to apply this framework. For instance, our prediction in the study of cancer, of the significant role of plasticity in conferring a selection advantage on TP53 mutations under micro-environmental pressure, still needs to be experimentally investigated. The parameter values used in this study are assumed to be chosen from a range of biologically relevant values (the source of a particular parameter value is given whenever it has been obtained from published work).

The approach taken in this paper may lead to a better understanding of the natural pathological mechanisms in the colonic/intestinal epithelium or any similar four—compartmental structures in ecology, population genetics, and social networks. The particular application of this approach to carcinogenesis may have some results at prognosis.

## Supporting information

S1 File Supplementary material. Appendix A—Characteristic equation, Appendix B—Fixation probability, Appendix C—Finding the quasi-fixed points and the associated survival probabilities, Appendix D—Phase diagram of the system.
(PDF)
