# Supplementary material for: Phenotypic heterogeneity in modeling cancer evolution
Source: PLoS One. 2017 Oct 30;12(10):e0187000. doi: 10.1371/journal.pone.0187000 (PMC5662227; doi:10.1371/journal.pone.0187000)
Supplement: S1 File — Appendix A—Characteristic equation, Appendix B—Fixation probability, Appendix C—Finding the quasi-fixed points and the associated survival probabilities, Appendix D—Phase diagram of the system. (PDF) [file pone.0187000.s001.pdf]

Supplementary material

Phenotypic heterogeneity in modeling cancer evolution

Ali Mahdipour-Shirayeh[1,*], Kamran Kaveh[2], Mohammad Koahandel[1,3], and Sivabal Sivaloganathan[1,3]

[1]Department of Applied Mathematics, University of Waterloo, Waterloo, ON N2L 3G1, Canada
[2]Program for Evolutionary Dynamics, Harvard University, Cambridge, MA 02138, USA
[3]Center for Mathematical Medicine, Fields Institute, Toronto, ON M5T 3J1, Canada
[*]Corresponding author. Email: amahdipo@uwaterloo.ca

ABSTRACT

The unwelcome evolution of malignancy during cancer progression emerges through a selection process in a complex heterogeneous population structure. In the present work, we investigate evolutionary dynamics in a phenotypically heterogeneous population of stem cells (SCs) and their associated progenitors. The fate of a malignant mutation is determined not only by overall stem cell and non-stem cell growth rates but also differentiation and dedifferentiation rates. We investigate the effect of such a complex population structure on the evolution of malignant mutations. We derive exactly calculated results for the fixation probability of a mutant arising in each of the subpopulations. The exactly calculated results are in almost perfect agreement with the numerical simulations. Moreover, a condition for evolutionary advantage of a mutant cell versus the wild type population is given in the present study. We also show that microenvironment-induced plasticity in invading mutants leads to more aggressive mutants with higher fixation probability. Our model predicts that decreasing polarity between stem and non-stem cells' turnover would raise the survivability of non-plastic mutants; while it would suppress the development of malignancy for plastic mutants. The derived results are novel and general with potential applications in nature; we discuss our model in the context of colorectal/intestinal cancer (at the epithelium). However, the model clearly needs to be validated through appropriate experimental data. This novel mathematical framework can be applied more generally to a variety of problems concerning selection in heterogeneous populations, in other contexts such as population genetics, and ecology.

Appendix A - Characteristic equation

Starting from the master equation of the given model (where for $N = N_{\rm S} + N_{\rm D}$, we assume that $1/N$ is the duration of each updating time)

$$\begin{aligned}\frac{\partial p(n_{\rm S},n_{\rm D};t)}{N\,\partial t} \;&=\; W_{\rm S}^{+}(n_{\rm S}-1,n_{\rm D})\,p(n_{\rm S}-1,n_{\rm D};t)+W_{\rm S}^{-}(n_{\rm S}+1,n_{\rm D})\,p(n_{\rm S}+1,n_{\rm D};t)\\ &+W_{\rm D}^{+}(n_{\rm S},n_{\rm D}-1)\,p(n_{\rm S},n_{\rm D}-1;t)+W_{\rm D}^{-}(n_{\rm S},n_{\rm D}+1)\,p(n_{\rm S},n_{\rm D}+1;t)\\ &-\big(W_{\rm S}^{+}(n_{\rm S},n_{\rm D})+W_{\rm D}^{+}(n_{\rm S},n_{\rm D})+W_{\rm S}^{-}(n_{\rm S},n_{\rm D})+W_{\rm D}^{-}(n_{\rm S},n_{\rm D})\big)\,p(n_{\rm S},n_{\rm D};t),\end{aligned} \tag{1}$$

the generating equation can be derived by assuming the probability generating function (PGF) in which coefficients define the probabilities of being at different possible states after a given time.

Starting from the master Eq. (1) discussed in the previous section, we can analyze the probability of absorption (fixation or extinction) using generating function techniques recently developed for the constant population birth-death processes[1]. Defining the probability generating function (PGF) of the probability density function $p(n_{\rm S},n_{\rm D};t)$ of having $n_{\rm S}$ mutant SCs and $n_{\rm D}$ mutant DCs at time t. Then the PGF is

$$F(z_{\rm S},z_{\rm D};t) = \sum_{n_{\rm S},n_{\rm D}} z_{\rm S}^{n_{\rm S}} z_{\rm D}^{n_{\rm D}}\, p(n_{\rm S},n_{\rm D};t) \tag{2}$$

Using the PGF one can rewrite the master equation (the latter equation) for PGF to derive the characteristic equations as follows

$$\frac{\partial F}{N\,\partial t} = (z_S^{-1}-1)\left\langle (W_S^- - z_S\,W_S^+)\,z_S^{n_S} z_D^{n_D}\right\rangle + (z_D^{-1}-1)\left\langle (W_D^- - z_D\,W_D^+)\,z_S^{n_S} z_D^{n_D}\right\rangle, \tag{3}$$

where the operators $W_S^{\pm}$ and $W_D^{\pm}$ are

$$\begin{aligned}
W_S^+(n_S,n_D) &= \mathrm{Prob}(n_S,n_D \to n_S+1,n_D)\\
&= \left(\frac{r_2\,(1-u_2)\,n_S + \tilde{r}_2\,\eta_2\,n_D}{r_1\,(N_S-n_S)+r_2\,n_S+\tilde{r}_1(N_D-n_D)+\tilde{r}_2\,n_D}\right)\frac{N_S-n_S}{N_S},\\
W_S^-(n_S,n_D) &= \mathrm{Prob}(n_S,n_D \to n_S-1,n_D)\\
&= \left(\frac{r_1\,(1-u_1)\,(N_S-n_S)+\tilde{r}_1\,\eta_1\,(N_D-n_D)}{r_1\,(N_S-n_S)+r_2\,n_S+\tilde{r}_1(N_D-n_D)+\tilde{r}_2\,n_D}\right)\frac{n_S}{N_S},\\
W_D^+(n_S,n_D) &= \mathrm{Prob}(n_S,n_D \to n_S,n_D+1)\\
&= \left(\frac{\tilde{r}_2\,(1-\eta_2)\,n_D + r_2\,u_2\,n_S}{r_1\,(N_S-n_S)+r_2\,n_S+\tilde{r}_1(N_D-n_D)+\tilde{r}_2\,n_D}\right)\frac{N_D-n_D}{N_D},\\
W_D^-(n_S,n_D) &= \mathrm{Prob}(n_S,n_D \to n_S,n_D-1)\\
&= \left(\frac{\tilde{r}_1\,(1-\eta_1)\,(N_D-n_D)+r_1\,u_1\,(N_S-n_S)}{r_1\,(N_S-n_S)+r_2\,n_S+\tilde{r}_1(N_D-n_D)+\tilde{r}_2\,n_D}\right)\frac{n_D}{N_D}.
\end{aligned} \tag{4}$$

$W_D^{\pm}(n_S,n_D)$ and $W_D^{\pm}(n_S,n_D)$ define the transition probabilities for increase/decrease in the number of mutant SCs and DCs respectively Acquiring these probabilities with those for no change in the number of mutant cells in either compartments, one can define the transition matrix which incorporates random walks among various states prior to fixation.

Substituting Eqs. (4) into generating equation (3), we obtain:

$$\begin{aligned}
\frac{\partial F}{N\,\partial t} &= \left\langle \frac{(z_S^{-1}-1)(r_2\,(1-u_2)\,n_S+\tilde{r}_2\,\eta_2\,n_D)(N_S-n_S)}{(r_1\,(N_S-n_S)+r_2\,n_S+\tilde{r}_1(N_D-n_D)+\tilde{r}_2\,n_D)\,N_S}\right\rangle\\
&+\left\langle \frac{(1-z_S)(r_1\,(1-u_1)\,(N_S-n_S)+\tilde{r}_1\,\eta_1\,(N_D-n_D))\,n_S}{N_S(r_1\,(N_S-n_S)+r_2\,n_S+\tilde{r}_1(N_D-n_D)+\tilde{r}_2\,n_D)}\right\rangle\\
&+\left\langle \frac{(z_D^{-1}-1)(\tilde{r}_2\,(1-\eta_2)\,n_D+r_2\,u_2\,n_S)(N_D-n_D)}{(r_1\,(N_S-n_S)+r_2\,n_S+\tilde{r}_1(N_D-n_D)+\tilde{r}_2\,n_D)\,N_D}\right\rangle\\
&+\left\langle \frac{(1-z_D)(\tilde{r}_1\,(1-\eta_1)\,(N_D-n_D)+r_1\,u_1\,(N_S-n_S))\,n_D}{(r_1\,(N_S-n_S)+r_2\,n_S+\tilde{r}_1(N_D-n_D)+\tilde{r}_2\,n_D)\,N_D}\right\rangle
\end{aligned} \tag{5}$$

Defining $\hat{n}_S = z_S\frac{\partial}{\partial z_S}, \hat{n}_D = z_D\frac{\partial}{\partial z_D}$, we conclude

$$\frac{\partial F}{N\,\partial t} = \left\{(z_S^{-1}-1)W_S^-(\hat{n}_S,\hat{n}_D)+(z_S-1)\,W_S^+(\hat{n}_S,\hat{n}_D)+(z_D^{-1}-1)W_D^-(\hat{n}_S,\hat{n}_D)+(z_P-1)W_D^+(\hat{n}_S,\hat{n}_D)\right\}F. \tag{6}$$

For large – N_S (N_D), we can simplify the latter equation for the probability generating function by keeping the linear derivative terms to leading order in N_S (N_D). This tends to

$$\begin{aligned}
\frac{\partial F}{N\,\partial t} &\simeq (z_S-1)\left\{\frac{r_2(1-u_2)\,z_S\,\frac{\partial F}{\partial z_S}+\tilde{r}_2\,\eta_2\,z_S\,\frac{\partial F}{\partial z_D}}{\hat{N}_r} - \frac{r_1\,(1-u_1)\,N_S\,\frac{\partial F}{\partial z_S}+\tilde{r}_1\,\eta_1\,N_D\,\frac{\partial F}{\partial z_S}}{\hat{N}_r\,N_S}\right\}\\
&+(z_D-1)\left\{\frac{\tilde{r}_2(1-\eta_2)\,z_D\,\frac{\partial F}{\partial z_D}+r_2\,u_2\,z_S\,\frac{\partial F}{\partial z_S}}{\hat{N}_r} - \frac{\tilde{r}_1\,(1-\eta_1)\,N_D\,\frac{\partial F}{\partial z_D}+r_1\,u_1\,N_S\,\frac{\partial F}{\partial z_D}}{\hat{N}_r\,N_D}\right\},
\end{aligned} \tag{7}$$

where the operator

$$\hat{N}_r = r_1\,(N_S-\hat{n}_S)+r_2\,\hat{n}_S+\tilde{r}_1(N_D-\hat{n}_D)+\tilde{r}_2\,\hat{n}_D, \tag{8}$$

can be considered constant when N_S and N_D are set to be large, $N_S, N_D \gg 1$.

Setting the coefficients of the derivatives $\frac{\partial F}{\partial z_S}$ and $\frac{\partial F}{\partial z_D}$ to zero, we obtain approximate quasi-stationary points of this constant population dynamics at the large–t limit. This relates to the corresponding martingales for $N_S, N_D \to \infty$ branching process limit. Denoting the solutions with $z_S^\star$ and $z_P^\star$, one attains

$$\begin{aligned} &(z_S^\star - 1)\,[r_2(1-u_2)\,z_S^\star - r_1(1-u_1) - \tilde{r}_1\,\eta_1] + (z_D^\star - 1)\,z_S^\star\,r_2\,u_2 = 0 \\ &(z_D^\star - 1)\,[\tilde{r}_2\,(1-\eta_2)\,z_D^\star - \tilde{r}_1\,(1-\eta_1) - r_1\,u_1] + (z_S^\star - 1)\,z_D^\star\,\tilde{r}_2\,\eta_2 = 0. \end{aligned} \tag{9}$$

Appendix B - Fixation probability

Taking advantage of the generating function, an exactly calculated approach for the fixation probability can be derived even in the presence of plasticity (when mutation and mutation–back do not occur). The results are obtained for a BD Moran process; however, a similar calculation can be performed for a Voter (DB) Model with presumably different fixed points for the same initial conditions. The boundary and initial conditions for the corresponding generating function are respectively as follows

$$F(z_S = 1, z_D = 1, t) = 1, \qquad \text{for any } t > 0, \tag{10}$$

$$F(z_S, z_D, t = 0) = z_S^i\, z_D^j, \tag{11}$$

where i and j are the initial number of cancer SCs and DCs respectively. For the following special cases, the system has two absorbing states at equilibrium which signify fixation and extinction for mutant cells in either compartments. We denote the probability of reaching extinction and fixation states by B_0 and B_1 respectively Thus we obtain the following result from the PGF at steady state

$$\begin{aligned} F(z_S, z_D, t\to\infty) &= p(n_S = 0, n_D = 0, t\to\infty) + p(n_S = N_S, n_d = N_D, t\to\infty)\, z_S^{N_S}\, z_D^{N_D} \\ &= B_0 + B_1\, z_S^{N_S}\, z_d^{N_D}, \end{aligned} \tag{12}$$

based on the boundary condition (10), $B_0 + B_1 = 1$. Biologically, since the mutant DCs are produced by cancer SCs, extinction of cancer SCs will result in replenishment of mutant DCs. Moreover, the co-operation between SCs and DCs suggests that it is impossible to have mutant SCs fixate while DCs become completely extinct. We conclude that

$$F(z_S, z_D, t\to\infty) = 1 - B_1\left(1 - z_S^{N_S}\, z_D^{N_D}\right). \tag{13}$$

Finally, applying the initial condition, $F(z_S, z_D, t = 0) = z_S^i\, z_D^j$ and the boundary condition, $F(z_S = 1, z_D = 1, t) = 1$, the fixation probability for an initial population of i malignant stem cells and j progenitors is derived as

$$\rho_{ij} = \frac{1 - (z_S^\star)^i\,(z_D^\star)^j}{1 - \left(z_S^\star\right)^{N_S}\left(z_D^\star\right)^{N_D}}, \tag{14}$$

where $(z_S^\star, z_D^\star)$ is the nontrivial fixed point of the generating Eq. (9).

Now one can conclude the fixation probability of a newborn mutant in the SC compartment, which means that in relation 14 ($i = 1$ and $j = 0$) to take over the entire population as follows:

$$\rho_S \equiv \rho_{10} = \frac{1 - z_S^\star}{1 - \left(z_S^\star\right)^{N_S}\left(z_D^\star\right)^{N_D}}. \tag{15}$$

On the other hand, one can obtain the fixation probability that an imposed malignant mutation in the DC compartment which eventually take over the whole population ($i = 0, j = 1$):

$$\rho_D \equiv \rho_{01} = \frac{1 - z_D^\star}{1 - \left(z_S^\star\right)^{N_S}\left(z_D^\star\right)^{N_D}}. \tag{16}$$

In general, the imposed mutation can occur randomly in the population. Assuming a uniform distribution for the occurrence of the mutation to give an equal chance to each of individuals within the population to get the malignant mutations. Then the average fixation probability of the mutant arising either in the SC or DC compartment is given by

$$\rho = \frac{1 - (N_S/N_{tot})z_S^\star - (N_D/N_{tot})z_D^\star}{1 - \left(z_S^\star\right)^{N_S}\left(z_D^\star\right)^{N_D}}. \tag{17}$$

with $N_{tot} = N_S + N_D$.

Appendix C - Finding the quasi–fixed points and the associated survival probabilities

An interesting scenario occurs when the normal component is not plastic, i.e. $\eta_1 = 0$, in which case the above equations can be simplified to the following expression of $z_{\rm S}^\star, z_{\rm D}^\star \neq 1$

$$z_{\rm D}^\star = \frac{\Big(r_1\,(1-u_2)\,(\tilde{r}_1 + r_1\,u_1)\Big)\,z_{\rm S}^\star + r_1\,(1-u_1)\,(\tilde{r}_1 + r_1\,u_1)}{\Big(r_1\,r_2\,(1-u_2)\,(1-\eta_1) - \tilde{r}_2\,r_2\,u_2\,\eta_2\Big)\,z_{\rm S}^\star - r_1\,r_2\,(1-u_1)\,(1-\eta_2)}. \tag{18}$$

Eqs. (**??**) can be reduced to the following closed form

$$\left(A - \frac{B}{z_{\rm S}^\star}\right)\left(E - \frac{F}{z_{\rm D}^\star}\right) = C\,.\,G, \tag{19}$$

where

$$\begin{aligned} &A = r_2\,(1-u_2), \quad B = r_1\,(1-u_1) + \tilde{r}_1\,\eta_1, \quad C = r_2\,u_2,\\ &E = \tilde{r}_2\,(1-\eta_2), \quad F = \tilde{r}_1\,(1-\eta_1) + r_1\,u_1, \quad G = \tilde{r}_2\,\eta_2. \end{aligned} \tag{20}$$

The latter equations together with the original system suggest the following solution for $z_{\rm S}$ and thus represent another fixed point of the problem:

$$\begin{aligned} (A^2E - ACG)\,(z_{\rm S}^\star)^3 + (ACF + C^2G + BCG + ACG - 2ABE - A^2E - ACE)\,(z_{\rm S}^\star)^2&\\ +(B^2E + BCE + 2ABE - BCG - BCF)\,z_{\rm S}^\star = B^2E.& \end{aligned} \tag{21}$$

Now let us consider the following particular cases which give rise to some interesting consequences of the model.

C1. Standard Moran process

Assume that there is no transition (migration) between SC and DC compartments (two islands), that is, $u_i, \eta_j \ll 1$ for $i, j = 1, 2$. Then each compartment will follow the mass–action BD Moran model with the fixed points for $z_{\rm S}^\star$ and $z_{\rm D}^\star$ where the overall behavior is akin to two disjoint Moran processes. The solutions to Eqs. (9) are

(1) $z_{\rm S}^\star = 1, z_{\rm D}^\star = 1,$

(2) $z_{\rm S}^\star = 1, z_{\rm D}^\star = \frac{\tilde{r}_1}{\tilde{r}_2},$

(3) $z_{\rm S}^\star = \frac{r_1}{r_2}, z_{\rm D}^\star = 1,$

(4) $z_{\rm S}^\star = \frac{r_1}{r_2}, z_{\rm D}^\star = \frac{\tilde{r}_1}{\tilde{r}_2}.$

where the solution (4) accounts for the fixed points of two separated Moran models in SC and Dc groups. Therefore, the fixation probabilities of starting from one imposed mutant in SC and DC groups respectively are

$$\rho_1 = \frac{1 - \frac{r_1}{r_2}}{1 - \left(\frac{r_1}{r_2}\right)^{N_{\rm S}}}, \qquad \rho_2 = \frac{1 - \frac{\tilde{r}_1}{\tilde{r}_2}}{1 - \left(\frac{\tilde{r}_1}{\tilde{r}_2}\right)^{N_{\rm D}}}. \tag{22}$$

Another interesting limit that results in a well-mixed Moran model in the SC class occurs when $\tilde{r}_{1,2} \simeq 0$ where SCs are also committed to reach a stationary state. Then the solutions to Eqs. (9) are given by

(1) $z_{\rm S}^\star = 1, z_{\rm D}^\star = 1,$

(2) $z_{\rm S}^\star = \frac{r_1}{r_2}, z_{\rm D}^\star = 1.$

Compared with the result from the previous case, the second solution relates to the solution of a well–mixed model occurring in the SC compartment where the only absorbing state for mutant DCs is to take over the whole population where the evolutionary scenario accounts for cancer DCs domination. In this scenario, the average fixation probability is

$$\rho = \frac{N_{\rm S}\left(1 - \frac{r_1}{r_2}\right)}{N_{\rm tot}\left(1 - \left(\frac{r_1}{r_2}\right)^{N_{\rm S}}\right)}, \tag{23}$$

C2. Invasion in hierarchical model (no plasticity)

Now we consider the case where no plastic potential is taken into account during the whole process, we assume that $r_1 = \tilde{r}_1 = 1$, $r_2 = \tilde{r}_2 = r$, $u_1 = u_2 = u$, and $\eta_1 = \eta_2 = 0$. in this situation, one obtains the following solutions

(1) $z_S^\star = 1, z_D^\star = 1,$

(2) $z_S^\star = \dfrac{1}{u_{\text{eff}}}, z_D^\star = 1$

(3) $z_S^\star = \frac{-r+u_1+u_2 u_1-1+u_2+\Gamma}{2r(-1+u_2)}, z_D^\star = \frac{u_1+1}{r}.$

where $u_{\text{eff}} = \frac{r(1-u_2)}{1-u_1}$ is the effective asymmetric division rate and

$$\Gamma^2 \quad = \quad 1 - 6ru_2u_1 + u_1^2 + 2ru_2 - 2r - 2u_2 - 2u_1 + 2ru_1 + r^2 + 2u_2u_1^2 + 2u_2^2u_1 + u_2^2 + u_2^2u_1^2. \tag{24}$$

The third solution cannot be maintained in reality since there is no cooperation between the compartments, which could result in support for the mutant SC by the mutant DC group. In such a case, the only possible outcome would be the fixation of the mutant DCs. Collectively, we find

$$\rho_S = \frac{1 - \frac{1-u_1}{r(1-u_2)}}{1 - \left(\frac{1-u_1}{r(1-u_2)}\right)^{N_S}}, \qquad \rho_D = 0, \qquad \rho = \frac{N_S}{N_{\text{tot}}}\rho_S. \tag{25}$$

C3. Invasion with phenotypic plasticity (dedifferentiation)

Suppose that dedifferentiation only occurs for the mutant DCs at a rate $\eta_2 = \eta$ for $\eta \gg \eta_1 \approx 0$. Also let assume that $r_1 = \tilde{r}_1 = 1$, $r_2 = \tilde{r}_2 = r$, $u_1 = u_2 = u$ and $\eta_2 = \eta$, then the solutions for $z_S^\star$ and $z_D^\star$ are

(1) $z_S^\star = 1, z_D^\star = 1,$

(2) $z_S^\star$ satisfies in the equation

$$(A^2E - ACG)\,(z_S^\star)^3 + (ACF + C^2G + BCG + ACG - 2ABE - A^2E - ACE)\,(z_S^\star)^2 \\ +(B^2E + BCE + 2ABE - BCG - BCF)\,z_S^\star = B^2E,$$

where $A = r(1-u), B = 1-u, C = ru, E = r(1-\eta), F = u+1, G = r\eta$, and $z_D^\star = \frac{(z_S^\star+1)(1-u^2)}{r[(1-u-ru\eta)z_S^\star-(1-u)(1-\eta)]}$.

The values for $z_S^\star$ and $z_D^\star$, in this case, satisfy the following relations:

$$\left(A - \frac{B}{z_S^\star}\right)\left(E - \frac{F}{z_D^\star}\right) = C.G, \qquad z_D^\star = \frac{(z_S^\star+1)(1-u^2)}{r[(1-u-ru\eta)z_S^\star - (1-u)(1-\eta)]}, \tag{26}$$

where $A = r(1-u), B = 1-u, C = ru, E = r(1-\eta), F = u+1, G = r\eta$.

The derived solutions introduce the possible fixed points of the characteristic equations. We investigate this case in more detail later and through analyzing the phase diagram of the generalized model for non-zero plasticity in the replicator dynamics.

Appendix D - Phase diagram of the system

Now, acquiring the replicator dynamics of the four compartment model which depict the alterations in the the average frequencies of mutant SCs and DCs, respectively. $x_S = \langle n_S(t)/N_S \rangle$ and $x_D = \langle n_D/N_D \rangle$, one obtains the following system of equations:

$$\begin{aligned} \frac{dx_S}{dt} &\approx \langle W_S^+ - W_S^- \rangle \\ &= \frac{[r_2(1-u_2) - r_1(1-u_1)]x_S(1-x_S) + \tilde{r}_2\eta_2 x_D(1-x_S) - \tilde{r}_1\eta_1 x_S(1-x_D)}{r_1(1-x_S) + r_2 x_S + \tilde{r}_1(1-x_D) + \tilde{r}_2 x_D}, \\ \frac{dx_D}{dt} &\approx \langle W_D^+ - W_D^- \rangle \\ &= \frac{[\tilde{r}_2(1-\eta_2) - \tilde{r}_1(1-\eta_1)]x_D(1-x_D) + r_2 u_2 x_S(1-x_D) - r_1 u_1 x_D(1-x_S)}{r_1(1-x_S) + r_2 x_S + \tilde{r}_1(1-x_D) + \tilde{r}_2 x_D}. \end{aligned} \tag{27}$$

At equilibrium, the fraction of mutant stem and non-stem cell groups would lead to a specific states which are the pseudo–fixed points of the problem. These type of fixed points can be attractive or repulsive, depending on the initial conditions of the system. As we described in the paper, according to the cooperation between mutant SCs and DCs (and similarly between normal SCs and DCs), the malignant individuals may become extinct or survive together. Now defining the fixed point as $(x_{\mathrm{S}}^{\star}, x_{\mathrm{D}}^{\star})$, it may tend either to $(0,0)$ or to $(1,1)$. Such a criteria suggests two distinct phases for the fate of the malignant cells which are separated by the phase boundary. At steady state, the following system can be derived for the fixed points $x_{\mathrm{S}}^{\star}$ and $x_{\mathrm{D}}^{\star}$:

$$\begin{aligned}
&[r_2(1-u_2) - r_1(1-u_1)]\, x_{\mathrm{S}}^{\star}(1-x_{\mathrm{S}}^{\star}) + \tilde{r}_2\,\eta_2\, x_{\mathrm{D}}^{\star}(1-x_{\mathrm{S}}^{\star}) - \tilde{r}_1\,\eta_1\, x_{\mathrm{S}}^{\star}(1-x_{\mathrm{D}}^{\star}) = 0,\\
&[\tilde{r}_2(1-\eta_2) - \tilde{r}_1(1-\eta_1)]\, x_{\mathrm{D}}^{\star}(1-x_{\mathrm{D}}^{\star}) + r_2\, u_2\, x_{\mathrm{S}}^{\star}(1-x_{\mathrm{D}}^{\star}) - r_1\, u_1\, x_{\mathrm{D}}^{\star}(1-x_{\mathrm{S}}^{\star}) = 0.
\end{aligned} \tag{28}$$

To analyze the latter system, we may consider two important cases:

Case 1. At first, we assume $u_1 = u_2 = \eta_1 = \eta_2 = 0$, then the solutions to the system (28) results in the extinction or fixation of mutant SCs and DCs. In this case, the phase diagram is simply divided to two advantageous $(r > 1)$ and disadvantageous $(r < 1)$ cases.

Case 2. Suppose that $r_1 = \tilde{r}_1 = 1, r_2 = \tilde{r}_2 = r, u_1 = u_2 = u, \eta_1 = 0$, and $\eta_2 =: \eta$. This introduces an interesting scenario in which plasticity occurs only for the cancerous cells but not for the WT individuals. These restrictions simplify system (28) to the following possible cases for $x_{\mathrm{S}}^{\star}$ and $x_{\mathrm{D}}^{\star}$

(1) $x_{\mathrm{S}}^{\star} = 0, \quad x_{\mathrm{D}}^{\star} = 0,$

(2) $x_{\mathrm{S}}^{\star} = 1, \quad x_{\mathrm{D}}^{\star} = 1,$

(3) $x_{\mathrm{S}}^{\star} = 1, \quad x_{\mathrm{D}}^{\star} = \frac{ru}{1-r+r\eta}$, and

(4) $x_{\mathrm{S}}^{\star} = r\,\eta\,\mathscr{A}\,\mathscr{M}_1^{-1}, \quad x_{\mathrm{D}}^{\star} = \mathscr{A}\,\mathscr{M}_2^{-1},$

where

$$\begin{aligned}
\mathscr{M}_1 &= 3ru^2 + 3r + 2u - 3r^2u^2 + 6r^2u - 6ru - r\eta - 2r^3u - 2r^2\eta u + r\eta u + 2r^2\eta\\
&\quad + r^3\eta u - 3r^2 + r^3 + r^3u^2 - r^3\eta - 1 - u^2,\\
\mathscr{M}_2 &= r^2u + r^2\eta - r^2 - 2ru - r\eta + 2r - 1 + u,\\
\mathscr{A} &= 2r - 1 - r\eta + r^2u + r^2\eta + u^2 - ru - r^2 + r\eta u - ru^2.
\end{aligned} \tag{29}$$

Among the given solutions (1)-(4) of the present case, the only acceptable non–trivial solution is (4). The solution (3) does not satisfy the condition $0 \leq x_{\mathrm{D}} \leq 1$ for all possible values of r, u, and η. Moreover, as we described above, the cooperation among mutant cells results in the fixation of both mutant SC and DC groups to the state $(1,1)$.

The last solution, then, implies that having $(x_{\mathrm{S}}, x_{\mathrm{D}}) \to (0,0)$ leads to the following solution

$$\mathscr{A} = (u + \eta - 1)r^2 + \left[-u^2 + (\eta - 1)u + 2 - \eta\right] r - 1 + u^2 = 0, \tag{30}$$

which characterizes the advantageous and disadvantageous regions for mutants (see Fig. 6 for more details). Another limit relates to the case where $(x_{\mathrm{S}}, x_{\mathrm{D}})$ approaches $(1,1)$. One obtains

$$r\,\eta = (r-1)(u-1) \tag{31}$$

This condition, which is not defined for $r > 1$, does not change the phase diagram and would not have any effect on the selection pressure of the system on mutants.

References

1. Houchmandzadeh B, Vallade M. The fixation probability of a beneficial mutation in a geographically structured population. New Journal of Physics. 2011;13(7):073020.
